# Polyextremotolerant lignocellulolytic bacteria from contrasting halo-alkaline habitats as sources of extremozymes for rice husk saccharification and biofuel applications

**DOI:** 10.3389/fmicb.2026.1982443

**Published:** 2026-10-28

**Authors:** Anjali Gupta, Shubham Pandey, Anannya Agrawal, Ashwini Chauhan, Kuo-Wei Huang, Ram Karan

**Affiliations:** 1 Department of Microbiology, University of Delhi South Campus, New Delhi, India; 2 Department of Biosciences and Technology, Dr. Vishwanath Karad MIT-World Peace University, Pune, India; 3 Center for Renewable Energy and Storage Technologies (CREST), KAUST Catalysis Center (KCC), Physical Science and Engineering Division, King Abdullah University of Science and Technology, Thuwal, Saudi Arabia

**Keywords:** alkaline pretreatment, enzymatic saccharification, extremophile, extremozymes, halotolerant microbes, lignocellulosic degradation, second-generation biofuels

## Abstract

**Rationale:**

Lignocellulosic agricultural residues represent an enormous and largely untapped feedstock for second-generation biofuels, yet their structural recalcitrance remains the central bottleneck for cost-effective bioconversion. Conventional mesophilic enzymes lose much of their activity under the high-salinity, alkaline and thermally stressful conditions that define industrial biomass processing- a gap that microorganisms from naturally halo-alkaline ecosystems are well positioned to fill. Rice husk, generated in massive quantities as a by-product of rice milling across South Asia, remains an underutilized substrate whose silica-rich, lignin-shielded architecture makes it an appropriate and demanding test case for halotolerant biocatalysts.

**Methods:**

Three ecologically distinct saline habitats were sampled: Poovar Lake, a brackish mangrove estuary in Kerala (pH 6.92, salinity (chloride concentration) 1.90 g/L), Varkala Beach, a high-chloride concentration coastal site (pH 8.24, salinity (chloride concentration) 31.16 g/L), and Sanjay Lake, an anthropogenic inland lake in Delhi (pH 9.25, salinity (chloride concentration) 0.42 g/L). Using selective enrichment on halophilic media at 40 °C across a salinity range of 3–9% (w/v) NaCl, 29 halotolerant isolates were recovered and screened for xylanase, cellulase, lipase, amylase and protease activity using plate-based zone assays. The four most active cellulase- and xylanase-producing isolates, identified by 16S rRNA gene sequencing as *Priestia flexa* (XBL51), *Bacillus proteolyticus* (XBL61), *Bacillus paralicheniformis* (XBL66) and “*Vibrio neocaledonicus*” (XBL71), were then evaluated for their ability to hydrolyze alkaline-pretreated rice husk (1.5% (w/v) NaOH, 60 °C, 90 min) under saline-alkaline stress (4.5% (w/v) NaCl, pH 8.5, 40 °C). Growth kinetics and reducing sugar release were monitored over 6-days, and post-degradation structural changes in the biomass were characterized by X-ray diffraction (XRD) and scanning electron microscopy (SEM).

**Results:**

Alkaline pretreatment significantly disrupted the crystalline architecture of rice husk, reducing the crystallinity index (CrI) from 49.31% to 33.93%, thereby loosening the structure and substantially improving substrate accessibility for enzymatic attack. Among individual isolates, monoculture of XBL61 and XBL71, as well as XBL51 + XBL66 + XBL71 showed the strongest growth on pretreated rice husk as the sole carbon source. Reducing sugar release across the top-performing isolates and consortia ranged from 1.69 to 1.97 mg/g over the 6-day incubation. Monocultures XBL61 and XBL66 emerged as the most effective isolates, exhibiting sustained sugar production. Among the two-isolate consortium, XBL51 + XBL71 exhibited synergistic growth and hydrolysis activity. Post-incubation, XRD analysis showed that XBL61 alone raised the CrI marginally to 52.10%, suggesting reduction of amorphous regions, including non-cellulosic components such as hemicellulose and amorphous cellulose. By contrast, the XBL51 + XBL71 consortium reduced the CrI to 49.07%, reflecting more extensive and balanced structural deconstruction. SEM corroborated these findings, revealing a clear progression from the intact, tightly laminated surface of untreated biomass through the fissured, loosened layers of the pretreated material, to the deeply porous, honeycombed morphology left following consortium-mediated degradation.

**Conclusion:**

These results demonstrate that halotolerant bacteria isolated from contrasting saline habitats, particularly when used as rationally designed consortia, can effectively deconstruct alkaline-pretreated rice husk under conditions that would substantially inhibit conventional mesophilic cellulases. The inherent stress tolerance of these isolates makes them promising candidates for lignocellulosic biorefinery processes where elevated salinity and alkaline pH are unavoidable operational realities. Enzyme-level characterization and fermentation coupling of these isolates represent logical next steps toward translating these findings into scalable platforms for second-generation biofuel production.

## Graphical abstract

**Figure:**
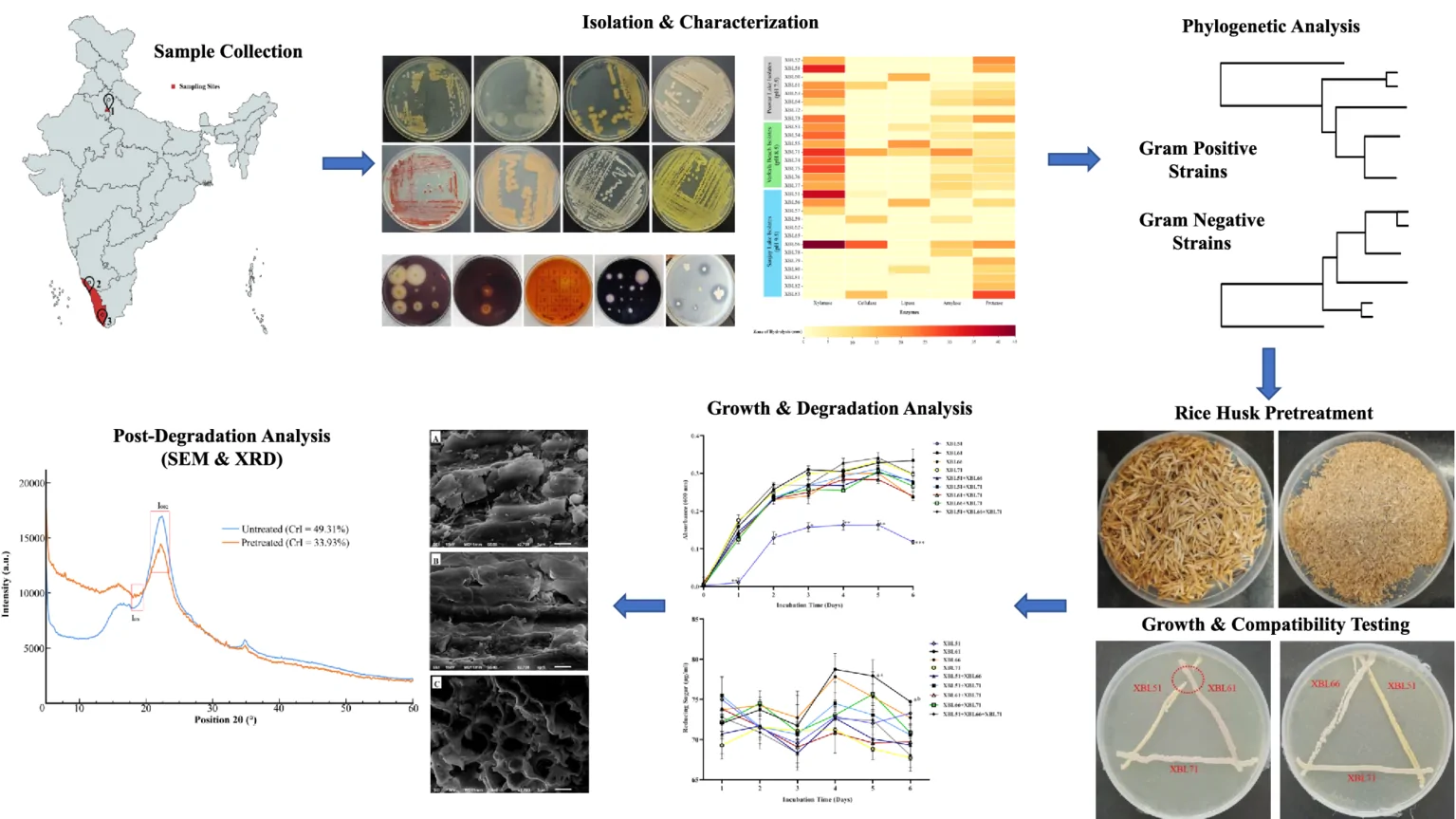

## 1 Introduction

The global population explosion, increasing industrialization and escalating energy demands have resulted in rapid depletion of fossil fuels, contributing to greenhouse gas emissions and climate change (Cherwoo et al., 2023; Wang and Azam, 2024). While first-generation biofuels offer a renewable alternative, their dependence on edible crops such as maize and sugarcane strains global food supplies and raises critical sustainability concerns (Cavelius et al., 2023). Consequently, second-generation biofuels derived from lignocellulosic biomass waste, including agricultural residues, forest products and mill waste, have emerged as a sustainable energy source. This form of biofuel provides the dual benefit of valorizing agricultural biowaste and producing renewable, carbon-neutral energy (Li and Takkellapati, 2018). However, large-scale commercialization remains hindered primarily by the inherent structural resistance of lignocellulosic biomass. Lignin acts as a robust physical barrier, restricting hydrolytic enzymes from accessing the targeted cellulose and hemicellulose substrates. Overcoming this barrier requires robust pretreatment strategies to enhance biomass accessibility for enzymatic hydrolysis. Furthermore, harsh physical and chemical pretreatment strategies often create an unfavorable processing environment, including high salinity, extreme pH and elevated temperatures, thereby reducing the catalytic efficiency of conventional mesophilic enzymes (Cheah et al., 2020; Hashemi et al., 2021).

The efficient enzymatic deconstruction of lignocellulose requires several synergistic enzymes, including laccases and peroxidases for lignin degradation (Jones and Solomon, 2015; Rivera-Hoyos et al., 2013), cellulases for cellulose hydrolysis (Mesa et al., 2016), xylanases for hemicellulose saccharification (Chaudhary et al., 2023), and amylases for hydrolysis of starch-based components (Bhatt et al., 2020). As the mesophilic variants exhibit limited efficiency under industrially relevant conditions, extremophilic and extremotolerant microorganisms offer a more robust alternative for bioprocessing (Grötzinger et al., 2018; Karan et al., 2012a; Karan et al., 2013; Karan et al., 2020; Kumar et al., 2012; Laye et al., 2017; Pandey et al., 2025a; Renn et al., 2021; Sysoev et al., 2021). Extremophiles secrete extremozymes that maintain structural stability and catalytic activity under conditions of high salinity, low water activity, extreme pH, extreme temperature profiles and other harsh conditions (Chettri et al., 2021; Kumar et al., 2012; Karan et al., 2012a; Karan et al., 2012b; Pandey et al., 2026a; Sysoev et al., 2021).

Recent research has increasingly focused on extremophilic isolates as sources of polyextremophilic enzymes, which combine multiple resilience traits. These robust biocatalysts represent efficient and sustainable solutions for the biofuel industry, where multiple physical and chemical stresses co-exist (Gupta et al., 2026; Pandey et al., 2026b). Such extremozymes demonstrate immense potential to efficiently saccharify lignocellulosic substrates into reducing sugars, thereby enabling the sustainable production of bioethanol, biogas, biobutanol and biodiesel within a circular bioeconomy framework (Pandey et al., 2025b).

In this study, rice husk was selected as the substrate on two counts: India generates roughly 22–24 million tonnes of it annually as a direct by-product of rice milling, and a large fraction of this is currently burned in open fields or disposed of without any value recovery, making it an available and underutilized feedstock. It’s unusually high silica content, combined with a tightly cross-linked lignin-hemicellulose matrix, also makes it one of the most recalcitrant agricultural residues to hydrolyze, which is precisely what makes it a meaningful substrate to test for any lignocellulolytic system. To maximize the availability of lignocellulosic biomass such as rice husk, for enhancing the overall efficiency of bioconversion, effective pretreatment strategies must be employed (Ale et al., 2026). Various physical, chemical, physicochemical and biological methodologies have been explored to disrupt the lignin matrix and increase cellulose accessibility for enzymatic degradation (Arce and Kratky, 2022; Brodeur et al., 2011; Gladysheva, 2025; Isroi et al., 2011).

Alkaline pretreatment was applied to rice husk as a preparatory step in this study before enzymatic hydrolysis, given its well-established effectiveness in disrupting the lignin-hemicellulose network and its relative simplicity and scalability compared with physical or combined physicochemical approaches. Alkaline pretreatment effectively promotes delignification by cleaving the ester bonds that cross-links lignin and carbohydrate molecules, thereby facilitating the selective removal of lignin from the biomass (Binod et al., 2010; Kaur et al., 2023; Shahabazuddin et al., 2018). This pretreatment process inherently generates an environment that is simultaneously alkaline and high in salinity. Consequently, polyextremotolerant microorganisms producing stable extremozymes that can function under extreme conditions are suitable for such saccharification applications.

Following pretreatment, enzymatic hydrolysis of the pretreated biomass was performed using the synergistic action of microbial cellulases and xylanases to liberate reducing sugars (Basera et al., 2024; Sukma et al., 2025). As hydrolysis efficiency is heavily affected by substrate accessibility, enzymatic activity and tolerance to inhibitory byproducts generated during pretreatment, extremophilic bacterial isolates offer an advantage over mesophilic counterparts. Their ability to produce stable, highly active extremozymes that function under harsh environmental conditions significantly enhances the overall lignocellulosic degradation process (Chettri et al., 2021). Further, the utilization of halotolerant and alkali-tolerant isolates offers significant advantages for *in vitro* saccharification. As chemical pretreatment generates a saline and alkaline environment, these isolates can operate directly within the residual biomass, preventing water-intensive neutralization steps while simultaneously suppressing mesophilic microbial contamination (Luo et al., 2022).

This led us to focus on prospecting halo- and alkali-tolerant bacteria from three ecologically contrasting saline/alkaline habitats, a brackish mangrove estuary, a marine coastal site and an anthropogenic inland lake, as a source of robust lignocellulolytic biocatalysts. Isolates were screened for hydrolytic enzyme production and characterized by 16S rRNA gene sequencing and phylogenetic analysis. The most active isolates were then tested directly on alkaline-pretreated rice husk under saline-alkaline conditions [4.5% (w/v) NaCl, pH 8.5], which substantially suppresses conventional mesophilic cellulases, with degradation performance evaluated using 6-day growth kinetics, saccharification assays, and post-hydrolysis structural characterization by XRD and SEM. The broader aim was to establish whether bacteria adapted to natural osmotic and pH stress carry practical value for lignocellulosic biorefineries, where such conditions are not exceptions but routine operating realities. Characterizing the enzyme repertoire of these isolates under industrially relevant stress conditions is central to establishing their utility as biocatalysts in a lignocellulosic biorefinery context.

## 2 Materials and methods

### 2.1 Sample collection sites and physicochemical characterization

For the isolation of halotolerant lignocellulose-degrading bacteria, three ecologically diverse sample collection sites were selected based on their distinct salinity (chloride concentration) profiles and the natural availability of complex plant biomass: Poovar Lake, Kerala (8°19′16.97″N 77°04′30.86″E), Varkala Beach, Kerala (8°44′07.19″N 76°42′10.70″E), and Sanjay Lake, Delhi (28°36′49.98″N 77°18′23.00″E). Water samples were collected from Poovar Lake, a brackish estuary bordered by mangroves, and Varkala Beach, a marine coastal site. A soil sample was collected from Sanjay Lake, an inland anthropogenic lake subjected to distinct seasonal osmotic stress. The samples were collected in sterile containers, and the physicochemical parameters of all samples were analyzed before processing. pH was recorded using an Eutech pH700 meter (Thermo Fisher Scientific), while the salinity (chloride concentration) was quantified using the Mohr titration method (Vogel et al., 1989). For the titration assay, 1 mL of 5% (w/v) potassium chromate was used as the indicator, and the samples were titrated against 0.1 M AgNO_3_. Poovar Lake water sample was analyzed directly, while the Varkala Beach water sample was diluted 1:10 in autoclaved Milli-Q water. For the soil sample (Sanjay Lake), a 1:5 (w/v) clarified aqueous extract was prepared for analysis by suspending 2 g of soil in 10 mL of autoclaved Milli-Q water.

### 2.2 Enrichment, isolation and biochemical characterization of microbes

Isolation of halotolerant bacteria was performed by directly spreading 100 μL of water samples on halophilic media agar plates (g/L) −20.0 MgSO_4_.7H_2_O, 0.3 CaCl_2_.2H_2_O, 2.0 KCl, 5.0 peptone, 10.0 yeast extract and 20.0 agar (Das et al., 2019) with varying salinity (3–9% w/v of NaCl). For the soil sample, 1 g of soil was inoculated in 100 mL of halophilic media broth to create a 1% (w/v) suspension, and was enriched for 16–18 h. Following enrichment, 100 μL of broth culture was spread onto halophilic media agar plates and incubated for 24–48 h. All the samples were incubated at 40 °C to reflect the industrial biomass saccharification conditions, selectively enriching thermo- and halotolerant microorganisms, while concurrently suppressing the dominant mesophilic background flora. Distinct colonies obtained on incubation were further streaked to obtain pure colonies. Gram staining and biochemical tests, including the indole test, MR-VP test, citrate utilization test, catalase test and oxidase test, were performed to characterize the isolates (Gupta et al., 2026). Purified bacterial isolates were regularly sub-cultured and preserved as glycerol stocks at −80 °C.

### 2.3 Evaluating Hydrolytic Enzyme Potential of the Isolates

Isolates were screened for xylanase, cellulase, lipase, amylase and protease activity using modified minimal agar media (g/L) −30.0 or 60.0 NaCl, 20.0 MgSO_4_.7H_2_O, 0.3 CaCl_2_.2H_2_O, 2.0 KCl, 1.0 yeast extract and 20.0 agar supplemented with specific substrates: 1% (w/v) corn cob xylan (xylanase), 1% (w/v) carboxymethyl cellulose (cellulase), 1% (v/v) palm oil with 0.01% (w/v) phenol red (lipase), 1% (w/v) starch (amylase) and 1% (w/v) skim milk powder (protease) (Gupta et al., 2026; Pandey et al., 2026a). While xylanase and cellulase are directly relevant to lignocellulose saccharification, the broader screening panel was included to map the full hydrolytic potential of the isolates. Amylase activity was assessed as starch-like components persist in some agricultural biomass fractions and their hydrolysis contributes to overall reducing sugar yield; lipase production was evaluated given the waxy cuticle layers present on rice husk surfaces, which can impede enzyme accessibility; and protease activity was profiled as an indicator of the general secretome diversity of these isolates, which is relevant to their adaptability across biorefinery applications where mixed-substrate valorization is increasingly sought (Laiba et al., 2026; Sethupathy et al., 2021). The isolates were spot-inoculated on screening media plates and incubated at 40 °C for 24–48 h in triplicate. To maintain physiologically relevant conditions, the screening medium for each isolate was adjusted to match the exact NaCl concentration and pH that supported optimal growth during initial isolation. Following incubation, hydrolytic zones were visualized by flooding the plates with Gram’s iodine for the xylanase, cellulase and amylase assays. For the lipase assay, phenol red acted as a pH indicator. In the protease assay, the halo zone around the colony was directly evaluated (Das et al., 2019; Saldarriaga-Hernandez et al., 2025). Based on the zones of hydrolysis produced by the isolates for cellulase and xylanase, four strains (XBL51, XBL61, XBL66, and XBL71) were selected, ensuring habitat-representative ecological diversity in the downstream screening panel.

For the quantitative assay, each of the selected isolate was inoculated into modified minimal media (g/L) −30.0 or 60.0 NaCl, 20.0 MgSO_4_.7H_2_O, 0.3 CaCl_2_.2H_2_O, 2.0 KCl, 1.0 yeast extract supplemented with 1% (w/v) corn cob xylan for xylanase production and 1% (w/v) carboxymethyl cellulose for cellulase production, and incubated at 40 °C for 24–48 h. The crude xylanase and cellulase activities were quantitatively determined at different temperatures, pH and NaCl concentrations, by measuring the release of reducing sugars using the 3,5-dinitrosalicylic acid (DNSA) method (Bailey et al., 1992; Miller, 1959).

### 2.4 16S rRNA-based identification of the selected hydrolase producers

16S rRNA gene sequencing was done to identify the bacterial isolates using colony PCR. The forward primer 27F (5′-AGAGTTTGATCMTGGCTCAG-3′) and reverse primer 1492R (5′-GGTTACCTTGTTACGACTT-3′) were used. The PCR conditions used were as follows: initial denaturation step at 95 °C for 3 min. This was followed by 29 cycles of denaturation at 95 °C for 40 s, annealing at 60 °C for 45 s and extension at 72 °C for 1 min and 40 s. The program concluded with a final extension step at 72 °C for 7 min, after which the samples were stored at 4 °C. The isolated 16S rRNA gene sequence was sequenced via Sanger sequencing at the Central Instrumentation Facility, University of Delhi South Campus. The obtained sequences ~630 to 1,500 bp were analyzed for taxonomic similarity using BLASTn (Basic Local Alignment Search Tool) against the NCBI and EzBioCloud databases^1^, taking EzBiocloud database as reference (Yoon et al., 2017) and subsequently deposited in the NCBI GenBank database^2^. For phylogenetic analysis, the sequences were aligned using MUSCLE in MEGA 12 software (Edgar, 2004; Kumar et al., 2024). Furthermore, the phylogenetic tree was constructed from closely related reference isolates using the Maximum Likelihood (ML) method in MEGA12, by utilizing the evolutionary models tailored to each bacterial group: the Tamura-Nei model (T93 + G + I) was utilized for the Gram-negative isolates (phylum *Pseudomonadota*), while the Kimura 2-parameter model (K2 + G + I) was applied to the Gram-positive isolates (phylum *Bacillota*). Both models incorporated a discrete Gamma distribution (+G) and allowed for a fraction of evolutionarily invariant sites (+I). To polarize the phylogenies, the Gram-positive clade was rooted using *Clostridium acetobutylicum* (NR 074511.2), and the Gram-negative clade was rooted using *Burkholderia cepacia* (NR 029209.1). Phylogenetic robustness was evaluated using a bootstrap analysis (1,000 replicates) with a ≥ 70% consensus threshold to account for the high conservation of 16S rRNA in these taxa. The final tree topologies were visualized using the Interactive Tree Of Life (iTOL) tool (Letunic and Bork, 2016).

### 2.5 Alkaline pretreatment of rice husk biomass

Rice husk (procured from Sonipat, Haryana) was utilized as the primary substrate. The rice husk was weighed and washed with water to remove adhering debris. Following this, it was dried in a hot air oven at 60 °C for 8–10 h and subsequently milled into a fine powder. The processed rice husk was stored in sealed zip-lock bags until further use (Kaur et al., 2023).

Alkali-based pretreatment was performed using modified conditions with 1.5% (w/v) NaOH at 60 °C for 90 min on the biomass (McIntosh and Vancov, 2011). A solid biomass loading ratio of 1:10 (w/v) was maintained, consisting of 5 g of rice husk suspended in 50 mL of 1.5% (w/v) NaOH solution (Sahu et al., 2025). Following pretreatment, the pretreated rice husk was cooled to room temperature and filtered through Whatman filter paper to separate the solid residue from the liquid fraction. The pretreated biomass was thoroughly washed with distilled water until it reached neutral pH, followed by drying for downstream enzymatic saccharification studies.

### 2.6 Substrate utilization and isolate compatibility test

The isolates were evaluated for their ability to utilize the raw agricultural residue; minimal media agar plates were prepared and supplemented with 1% (w/v) rice husk as the sole carbon source (Chang et al., 2024). The plates were streaked with the selected isolates and incubated at 40 °C until visible growth was observed. *E. coli* was used as a control.

Subsequently, a cross-streak compatibility test was performed to ensure the synergistic co-existence of the selected isolates before consortium development. The respective isolates were inoculated via intersecting streaks on halophile media agar plates, incubated at 40 °C for 24–48 h. Compatibility was determined by the absence of clearance zones or growth suppression at the interfacial boundaries of the streak lines. Isolates that exhibited uniform, uninhibited growth at the intersections were deemed mutually compatible (Manasa et al., 2021). These compatible isolates and their specific consortium combinations were consequently selected for subsequent enzymatic saccharification and substrate degradation experiments.

### 2.7 Assessment of lignocellulolytic activity: growth, saccharification and structural analysis

#### 2.7.1 Assessment of lignocellulolytic efficiency via growth and saccharification analysis

To evaluate the lignocellulose-degrading efficiency of the isolates, the selected isolates were inoculated into minimal medium at pH 8.5 and 4.5% (w/v) NaCl, supplemented with 4% (w/v) pretreated rice husk as the sole carbon source (Ire et al., 2016). The total initial inoculum was strictly standardized across all treatments. Overnight cultures of each isolate were normalized to an OD_600_ of 1.0, and the final total inoculum concentration was maintained at 1% (v/v) for all flasks. Monocultures were inoculated with 1% (v/v) of the respective isolate. For microbial consortia, the normalized suspensions were combined in equal proportions to achieve the same 1% total volume: two-isolate consortia received 0.5% (v/v) of each isolate, and three-isolate consortia received 0.33% (v/v) of each isolate, ensuring an equivalent initial cellular biomass. An uninoculated medium served as the blank, while *E. coli* was utilized as a negative control. The cultures were incubated at 40 °C with continuous agitation at 170 rpm for 6 days. Microbial growth and the subsequent release of reducing sugars were monitored every 24 h by taking a 1 mL sample. Briefly, to assess microbial proliferation, OD_600_ was measured in triplicate for 100 μL of each sample in a 96-well microplate using a microplate reader (SkanIt Software version 7.1). The remaining 900 μL of the sample was centrifuged at 10,000 rpm for 10 min to pellet the microbial cells and residual biomass. The resulting cell-free supernatant was then subjected to the DNSA assay (in triplicate) to quantify the concentration of released reducing sugars by measuring absorbance at 540 nm (Oke et al., 2017).

#### 2.7.2 Characterization of degraded lignocellulosic biomass via SEM and XRD

The structural and crystallographic changes that occurred during the hydrolysis of the lignocellulosic biomass were evaluated following the 6-day incubation. The solid residues from the best-performing cultures were harvested, washed with deionized water to remove the residual culture media and cells and dried at 60 °C overnight.

To determine changes in the crystallinity of the cellulose structure following pretreatment and microbial degradation, the samples were subjected to X-ray diffraction (XRD). The diffractograms for untreated, alkaline-pretreated, and inoculated samples were recorded using the XRD facility (Rigaku SmartLab) at the University Science Instrumentation Center (USIC) of the University of Delhi. The analysis was performed over a 2*θ* range of 5° to 60° at a continuous scan rate of 2°/min (Hafid et al., 2021; Oke et al., 2017). The resulting diffraction profiles were subsequently used to calculate and compare the Crystallinity Index (CrI) for the untreated, pretreated and hydrolyzed biomass samples.

Further, to analyze the structural and topological changes on the surface, the rice husk samples were examined using Scanning Electron Microscopy (SEM) at the University Science Instrumentation Center (USIC), University of Delhi. The dried biomass samples were sputter-coated with a thin layer of gold prior to observation under the scanning electron microscope (JEOL JSM-6610LV) (Oke et al., 2017).

### 2.8 Statistical analysis

The growth kinetics and saccharification assays were performed in biological triplicate. Data were expressed as the mean ± standard deviation. Statistical significance between the experimental groups over the incubation period was determined using Two-Way Repeated Measures (RM) ANOVA. The Geisser–Greenhouse correction was applied to account for lack of sphericity, followed by Tukey’s multiple comparisons test to evaluate pairwise differences. Statistical significance was defined as *p* < 0.05. Analysis was performed using GraphPad Prism version 8.0.2 for Windows (GraphPad Software, Boston, Massachusetts USA, www.graphpad.com).

## 3 Results and discussion

### 3.1 Isolation, morphological and biochemical characterization of halotolerant isolates

Three distinct sites were selected for sample collection. The location and physico-chemical parameters of the collected samples were measured (Supplementary Table S1). About 29 distinct alkali- and halotolerant bacterial isolates were isolated from these sites. Of these 29 isolates, 8 isolates were obtained from Poovar Lake, 8 isolates from Varkala Beach and 13 isolates from Sanjay Lake. The sample was inoculated in halophilic media containing various salt concentrations ranging from 3% to 9% (w/v) NaCl, however no growth was observed above 6% (w/v) NaCl. This threshold reflects the natural physicochemical limits of the sampling site encompassing marine, brackish and inland environments. As these habitats lack hypersaline selection pressure, the indigenous microbial communities are evolutionarily adapted for halotolerance rather than extreme halophily, lacking the extensive osmoregulatory machinery required to survive salt concentrations exceeding 6% (w/v) NaCl. This distinction is industrially advantageous since it indicates that these isolates can function effectively at moderate salinities generated during alkaline biomass pretreatment without requiring extreme conditions that may complicate downstream fermentation.

The morphological characteristics of all isolates, including colour, size, shape, texture, margin and elevation are listed in Supplementary Table S2. The colony colour of the obtained isolates included white, cream, yellow, orange and red, while colony size ranged from punctiform to large (Supplementary Figure S1). Most isolates exhibited irregular morphology with undulate margins and raised or convex elevations. Colony texture varied from smooth and rough to mucoid. Similarly, the results of Gram staining and biochemical tests are summarized in Supplementary Table S3. Microscopic examination revealed that most isolates were rod-shaped, while some were cocci.

Additionally, Gram-positive bacteria constituted a major proportion of the isolates, particularly those isolated from Poovar Lake and Sanjay Lake. Biochemical characterization demonstrated metabolic diversity among the isolates. Most isolates were catalase positive, indicating their ability to detoxify reactive oxygen species (ROS) generated under stressful conditions. Oxidase activity was also observed in many isolates, indicating the presence of cytochrome c oxidase in their respiratory pathways (Hederstedt, 2022). The methyl red (MR) test was positive for a majority of the isolates, indicating mixed-acid fermentation.

In contrast, only a few isolates tested positive for the Voges-Proskauer (VP) test, highlighting variability in glucose metabolic pathways. Citrate utilization was observed in a few isolates, demonstrating their ability to utilize citrate as the sole carbon source. All isolates exhibiting distinct phenotypic characteristics were designated as XBL (Xtremophiles Biotech Lab) isolates for further study.

### 3.2 Hydrolytic enzyme activity profile of the isolates

All 29 isolates were qualitatively screened for xylanase, cellulase, amylase, lipase and protease activities using preliminary activity by plate assay (Supplementary Figure S2). Majority of isolates tested positive for xylanase and protease production. Several isolates also exhibited positive results for amylase activity. Conversely, fewer isolates demonstrated detectable cellulase and lipase activities. Based on the qualitative screening visualized in the heatmap (Figure 1), a higher proportion of isolates recovered from Varkala Beach exhibited prominent zones of hydrolysis across multiple enzyme classes, suggesting a robust broad-spectrum activity. In contrast, isolates from Sanjay Lake generally displayed fewer positive qualitative hits for broad hydrolase activity. Xylanase activity was frequently observed in isolates growing at pH 8.5, particularly among those from Varkala Beach. On the other hand, isolates at pH 9.5 from Sanjay Lake demonstrated distinct and substantial zones of hydrolysis for both xylanase and cellulase, such as in case of XBL51 and XBL66. This ability to secrete active hydrolytic enzymes at highly alkaline pH is particularly advantageous for application in biofuel processing, where alkaline pretreatment elevates the pH of the substrate prior to enzymatic saccharification.

**Figure 1:**
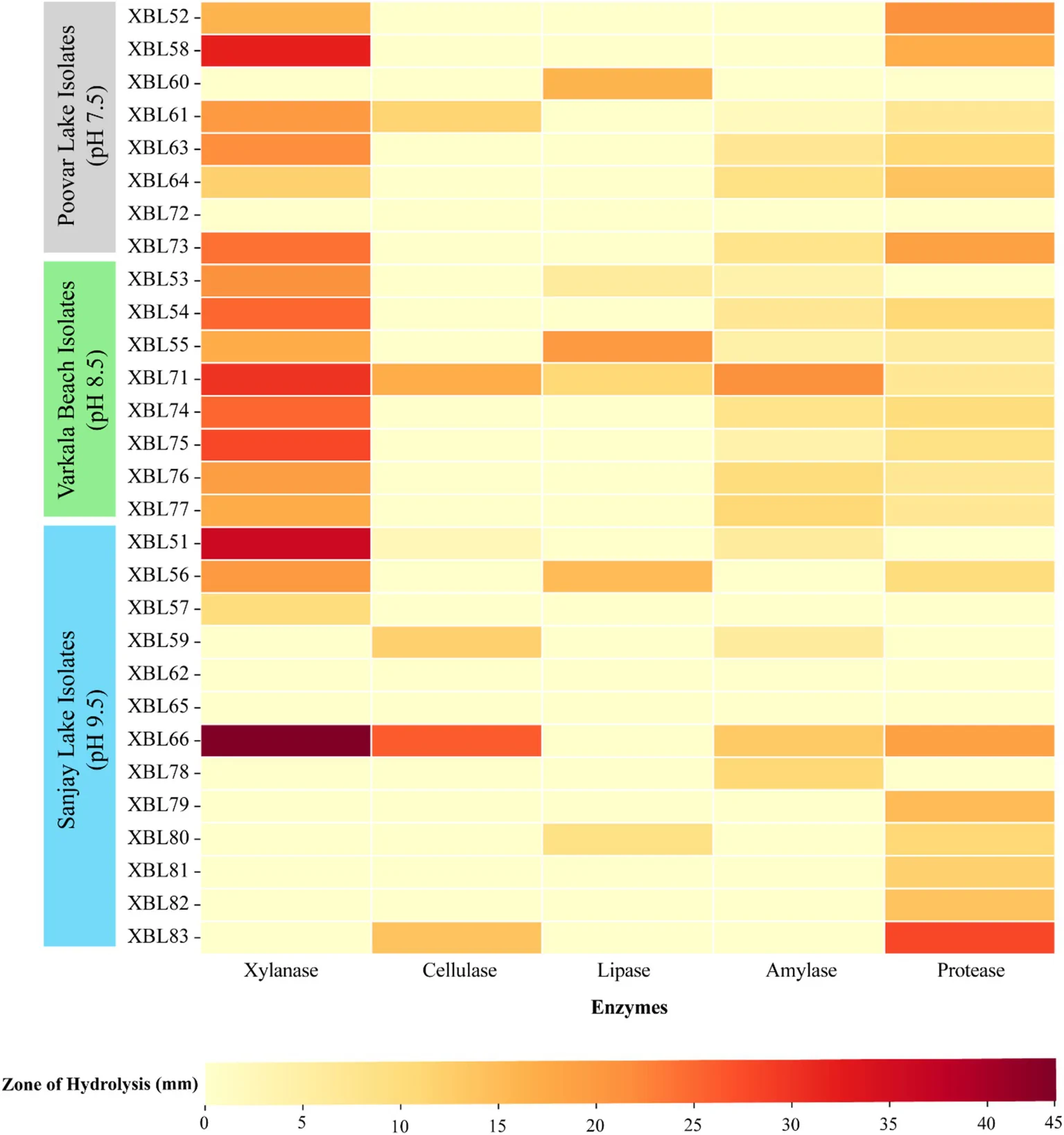
Enzyme screening of halotolerant isolates: zone of hydrolysis (mm). Heatmap depicting the hydrolytic enzyme activity profiles of 29 halotolerant bacterial isolates. Enzyme production was evaluated via plate-based zone of clearance assays conducted at the native isolation pH of each isolate (pH 7.5, gray; pH 8.5, green; pH 9.5, blue). Each row represents a single isolate at its respective pH at which the enzyme assay was conducted, and each column corresponds to one of five enzymes (xylanase, cellulase, lipase, amylase, protease). Colour intensity reflects the diameter of the hydrolysis zone, ranging from pale yellow (minimal or no activity) to deep red/maroon (maximum activity). Isolates are grouped by sampling site: Poovar Lake (pH 7.5), Varkala Beach (pH 8.5), and Sanjay Lake (pH 9.5).

The hydrolytic activity across multiple isolates at pH 8.5–9.5 strengthens their potential for application in post-alkaline pretreatment saccharification steps, where pH neutralization is often incomplete or deliberately avoided to reduce processing costs. XBL51, XBL58, XBL66, XBL71 and XBL75 were found to be among the best xylanase producers, with hydrolysis zones of 36.0 mm, 32.1 mm, 43.5 mm, 30.2 mm and 28.0 mm, respectively. For the cellulase enzymes, XBL66, XBL71 and XBL83 produced maximum hydrolysis zones of 26.1 mm, 17.4 mm and 14.0 mm, respectively. For lipase, amylase and protease, the best producers were XBL55 (20.2 mm), XBL71 (21.1 mm) and XBL83 (28.0 mm) respectively (Supplementary Table S4). From the 29 screened halotolerant isolates, the isolates (XBL51, XBL66 and XBL71) exhibiting the most prominent zones of hydrolysis for cellulase and xylanase, were selected for further characterization and application in lignocellulose degradation assays. XBL61 was additionally selected as the only Poovar Lake isolate demonstrating concurrent positive activity for both cellulase and xylanase, ensuring habitat-representative ecological diversity in the downstream screening panel.

Further, the quantitative characterization of xylanase and cellulase was carried out for the selected isolates (XBL51, XBL61, XBL66 and XBL71). The quantitative assays revealed good xylanase activity across moderate-to-high temperature range (30–50 °C). Also, the enzymes were found to be active at a pH range of 7.0–10.0 and over a salinity range of 3%–9% (w/v) NaCl. On the other hand, the cellulase activity was observed over 20–50 °C temperature, 6.0–10.0 pH and a salinity range of 3%–12% (w/v) NaCl, thus indicating the catalytic efficiencies of these polyextremotolerant enzymes under various harsh conditions.

### 3.3 Molecular identification and phylogenetic analysis of the isolates

16S rRNA gene sequencing was performed to identify selected isolates based on their hydrolytic enzyme activity profiles. This was followed by initial taxonomic analysis via the BLASTn (Basic Local Alignment Search Tool) against the NCBI database and the EzBioCloud type strain database (http://www.ncbi.nlm.nih.gov/; https://www.ezbiocloud.net/) to determine the closest strain resembling our selected XBL isolates (Table 1). The isolates showed high sequence similarity to recognized bacterial taxa, with sequence identities ranging from 98.25% to 100.00%.

**Table 1:** Molecular identification of selected halotolerant isolates based on 16S rRNA gene sequence comparison using EzBioCloud database, with corresponding GenBank accession numbers.

XBL series	EzBiocloud closest match	% Similarity (EzBiocloud)	NCBI accession no.
Poovar lake isolates			
XBL52	*Fictibacillus rigui*	99.45	PV652634
XBL58	*Bacillus xiamenensis*	99.46	PV652630
XBL60	*Rossellomorea marisflavi*	99.88	PV652637
XBL61	*Bacillus proteolyticus*	99.33	PV652629
XBL63	*Priestia megaterium*	99.67	PZ413221
XBL64	*Bacillus tequilensis*	99.27	PZ413224
Varkala beach isolates			
XBL53	*Vibrio coralliilyticus*	98.25	PV652639
XBL54	*Alteromonas macleodii*	100.00	PV652628
XBL55	*Marinobacter litoralis*	99.29	PV652638
XBL71	“*Vibrio neocaledonicus*”	99.50	PZ520481
Sanjay lake isolates			
XBL51	*Priestia flexa*	99.88	PV644243
XBL56	*Mammaliicoccus sciuri*	98.84	PV652636
XBL57	*Enterobacter nematophilus*	99.87	PV652632
XBL59	“*Halobacillus marinus*”	100.00	PV652633
XBL62	*Leclercia pneumoniae*	99.65	PV652635
XBL65	*Enterobacter nematophilus*	99.16	PZ413226
XBL66	*Bacillus paralicheniformis*	99.78	PZ413323

Of all the identified isolates, XBL51 showed 99.88% similarity to *Priestia flexa,* XBL61 showed 99.33% similarity to *Bacillus proteolyticus,* XBL66 showed 99.78% similarity to *Bacillus paralicheniformis* and XBL71 showed 99.50% similarity to “*Vibrio neocaledonicus*”.

Phylogenetic reconstruction based on 16S rRNA gene sequences revealed a diverse taxonomic distribution among the isolated lignocellulolytic bacteria. Utilizing the Maximum Likelihood method, the isolates were broadly divided into distinct Gram-positive and Gram-negative clades, with *Clostridium acetobutylicum* NR 074511.2 and *Burkholderia cepacia* NR 029209.1 serving as the outgroup, respectively, (Figure 2). The robust tree topology, supported by high bootstrap values (≥70%), indicates the acceptable identification and evolutionary divergence of the screened isolates.

**Figure 2:**
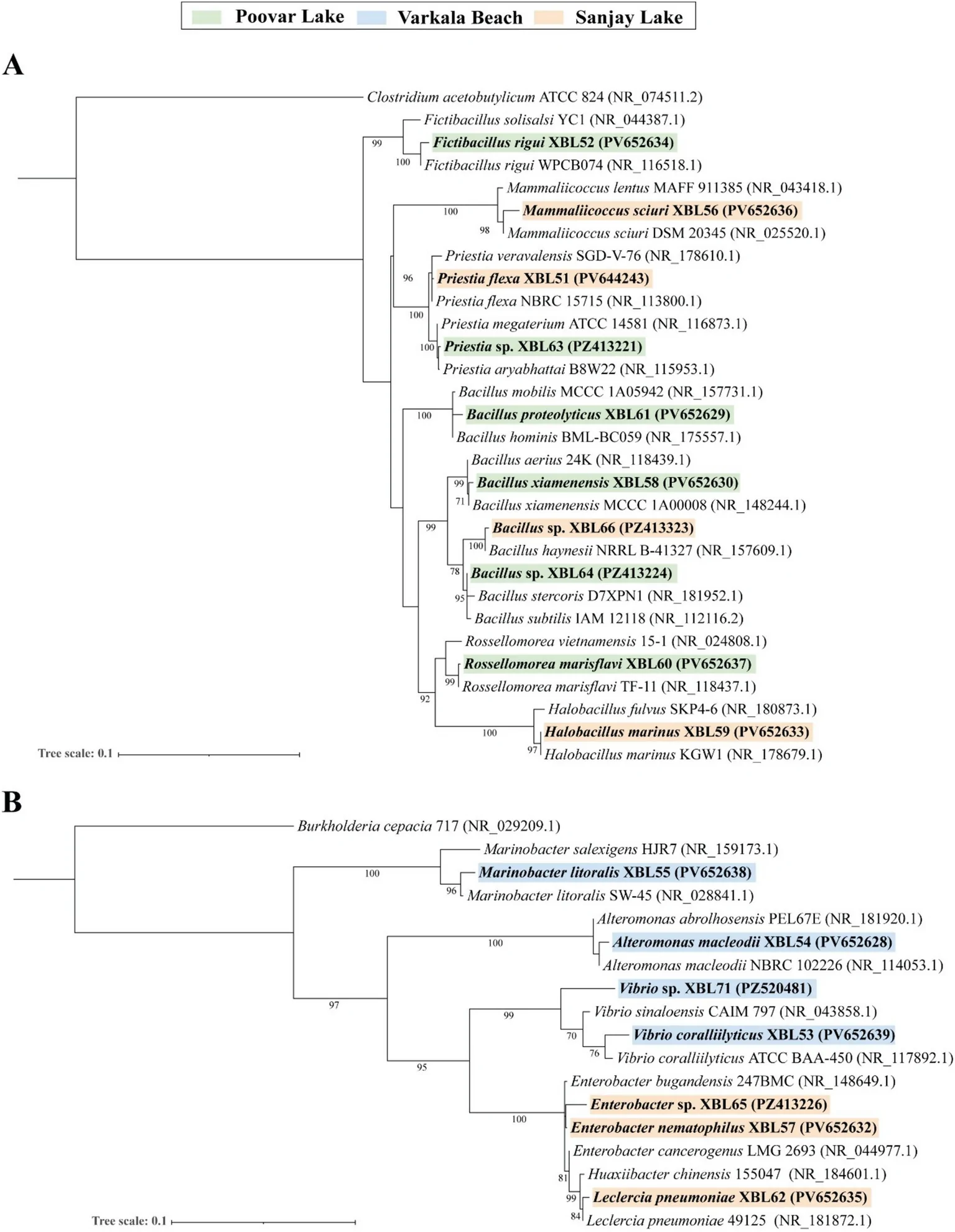
Phylogenetic reconstruction of representative lignocellulolytic bacterial isolates based on 16S rRNA gene sequences. Evolutionary relationships were inferred using the maximum likelihood method with bootstrap analysis based on 1,000 replicates; bootstrap values ≥70% are displayed at the nodes. **(A)** Phylogenetic clade comprising the Gram-positive isolates (phylum *Bacillota*), tree was rooted using *Clostridium acetobutylicum* NR 074511.2. **(B)** Phylogenetic clade comprising the Gram-negative isolates (phylum *Pseudomonadota*), the tree was rooted using *Burkholderia cepacia* NR 029209.1. The scale bar indicates the number of nucleotide substitutions per site.

The Gram-positive isolates were mapped to the phylum *Bacillota* (Figure 2A), exhibiting significant diversity, including isolates from the species *Mammaliicoccus*, *Fictibacillus*, *Priestia*, *Bacillus*, *Rossellomorea* and *Halobacillu*s. The inclusion of the halophilic genus *Halobacillus* (XBL59) further emphasizes the extremotolerant nature of the isolates. On the other hand, the Gram-negative isolates clustered predominantly within the phylum *Pseudomonadota* (Figure 2B), demonstrating strong phylogenetic affinities with the marine and environmental genera. The tree clearly defines well-supported subclades for *Marinobacter*, *Alteromonas*, *Vibrio*, *Enterobacter* and *Leclercia* species. Further, the presence of genera such as *Marinobacter* (XBL55) and *Alteromonas* (XBL54) within this clade strongly establishes the successful bioprospecting of halotolerant taxa capable of functioning in saline bioprocessing environments. Equally noteworthy is the placement of XBL71 within the *Vibrio* clade, showing 99.50% sequence similarity to “*Vibrio neocaledonicus*.” The genus *Vibrio* is overwhelmingly associated in the literature with marine pathogenicity and chitin degradation. Cellulolytic and xylanolytic activity in *Vibrio* species is far less commonly reported. Demonstration of xylanase and cellulase activity by these, tends to occur in coastal and estuarine isolates where plant-derived lignocellulosic material regularly enters the marine environment via riverine inputs and tidal exchange (Bamrungpanichtavorn et al., 2023; Li et al., 2020; Ren et al., 2021). XBL71 was isolated from Varkala Beach, a high-salinity coastal site, which places it in an environment where terrestrial plant detritus mixes with saline water, creating selection pressure for organisms capable of degrading both chitin and structural plant polysaccharides. Its xylanase and cellulase activity, confirmed by plate assay, is therefore ecologically coherent even if taxonomically unexpected.

### 3.4 Substrate utilization on pretreated rice husk

Before committing the selected isolates to liquid-phase saccharification assays, it was important first to confirm that they could actively grow on pretreated rice husk as the sole available carbon source, thus distinguishing actual lignocellulose utilizers from isolates that might tolerate its presence. All four selected isolates, XBL51, XBL61, XBL66 and XBL71, produced visible growth on minimal media agar plates supplemented with 1% (w/v) alkaline-pretreated rice husk, suggesting their potential capacity to utilize the carbohydrate components of the biomass (Supplementary Figure S3). The negative control, *E. coli*, showed negligible growth under these conditions, as expected, given its inability to produce the necessary lignocellulolytic enzymes to break down structural polysaccharides. The selected isolates demonstrated sustained growth and colonization when cultured with the structurally complex rice husk, suggesting functional substrate utilization. This indicates that the isolates are not merely producing hydrolases in the presence of a soluble inducer, instead possess definitive capacity for active lignocellulose depolymerization which was subsequently demonstrated through the quantitative saccharification assays in Section 3.6.

### 3.5 Compatibility testing and rational design of microbial consortia

After establishing that individual isolates can utilize and grow on lignocellulose as a primary carbon source, we studied whether they can act cooperatively as a consortium for lignocellulosic degradation. Microbial consortia are often found to outperform monocultures in lignocellulose degradation as different organisms contribute complementary enzyme activities, enabling a synergistic deconstruction of the substrate in comparison to what any single organism can achieve. However, this holds true, only when the isolates can co-exist without suppressing each other’s growth. To ensure this, a cross-streak compatibility test was performed for all combinations of the selected four isolates (Supplementary Figure S4; Table 2).

**Table 2:** Compatibility test between XBL51, XBL61, XBL66 and XBL71.

Consortia isolates	Results
XBL51 + XBL61	−
XBL51 + XBL66	+
XBL51 + XBL71	+
XBL61 + XBL66	−
XBL61 + XBL71	+
XBL66 + XBL71	+
XBL51 + XBL61 + XBL66	−
XBL51 + XBL61 + XBL71	−
XBL51 + XBL66 + XBL71	+
XBL61 + XBL66 + XBL71	−

(+) Compatible: no zone of inhibition at the intersection; (−) incompatible: zone of inhibition observed at the streak junction, indicating active growth suppression.

Among the two-isolate combinations, XBL51 + XBL66, XBL51 + XBL71, XBL61 + XBL71 and XBL66 + XBL71 all showed uninhibited, confluent growth at their intersection points, indicating genuine compatibility and the absence of antagonistic interactions. In contrast, XBL51 + XBL61 and XBL61 + XBL66 both produced clear zones of inhibition at the streak junctions, indicating that XBL61 actively suppresses the growth of these two partners. While this suppression could be due to non-specific factors like nutrient competition or pH shifts, it strongly aligns with what is known about *Bacillus proteolyticus*, the closest relative of XBL61, since members of the genus *Bacillus* are well-documented producers of antimicrobial lipopeptides such as iturin, fengycin and surfactin, as well as bacteriocins that can inhibit closely related organisms sharing the same ecological niche (Fira et al., 2018; Stein, 2005; Yu et al., 2022).

Further, XBL61 was found to be compatible with XBL71 but not with XBL51 or XBL66 suggesting its competitive/inhibitory activity may be isolate-specific rather than broad-spectrum, a characteristic worth exploring in further work on the enzymatic and secondary metabolite profiles of this isolate. What further strengthens this interpretation is the taxonomic pattern of the inhibition itself: both suppressed partners, XBL51 (*Priestia flexa*) and XBL66 (*Bacillus paralicheniformis*), are Gram-positive isolates, while the compatible partner, XBL71 (“*Vibrio neocaledonicus*”), is Gram-negative. This Gram-positive-selective antagonism is a well-recognized signature of bacteriocin activity in *Bacillus* species, in which such compounds preferentially target phylogenetically related competitors rather than more distantly related organisms (Abriouel et al., 2011; Stein, 2005). Genome mining for biosynthetic gene clusters or cell-free supernatant inhibition assays in future work would help confirm the identity of the active compound and could reveal whether XBL61 holds value beyond lignocellulose degradation as a source of narrow-spectrum antimicrobial agents.

In the three-isolate combinations, the pattern held consistently: every consortium involving XBL61: XBL51 + XBL61 + XBL66, XBL51 + XBL61 + XBL71 and XBL61 + XBL66 + XBL71, showed incompatibility, while XBL51 + XBL66 + XBL71 remained fully compatible. These compatibility outcomes directly guided the selection of consortia for downstream saccharification experiments.

### 3.6 Growth kinetics and reducing sugar release during rice husk saccharification

To assess the ability of microbes to degrade the pretreated rice husk, their growth and reducing sugar production were monitored over a 6-day incubation period. The growth of both individual isolates and their consortia was studied on alkaline pretreated lignocellulosic biomass in minimal media. Uninoculated control flasks were utilized as blanks to normalize optical density readings, and nullify the insoluble biomass that can cause background turbidity. Furthermore, the negative biological control (*E. coli*) showed negligible changes in optical density over the incubation period. The gradual increase in normalized absorbance for the cultures at 600 nm throughout the incubation period, coupled with the synchronous release of reducing sugars, highlights the ability of these isolates to utilize the lignocellulosic components of the rice husk as their primary carbon source (Figure 3). Maintaining this slightly alkaline environment also streamlined the biochemical workflow by reducing neutralization requirements post-alkaline pretreatment.

**Figure 3:**
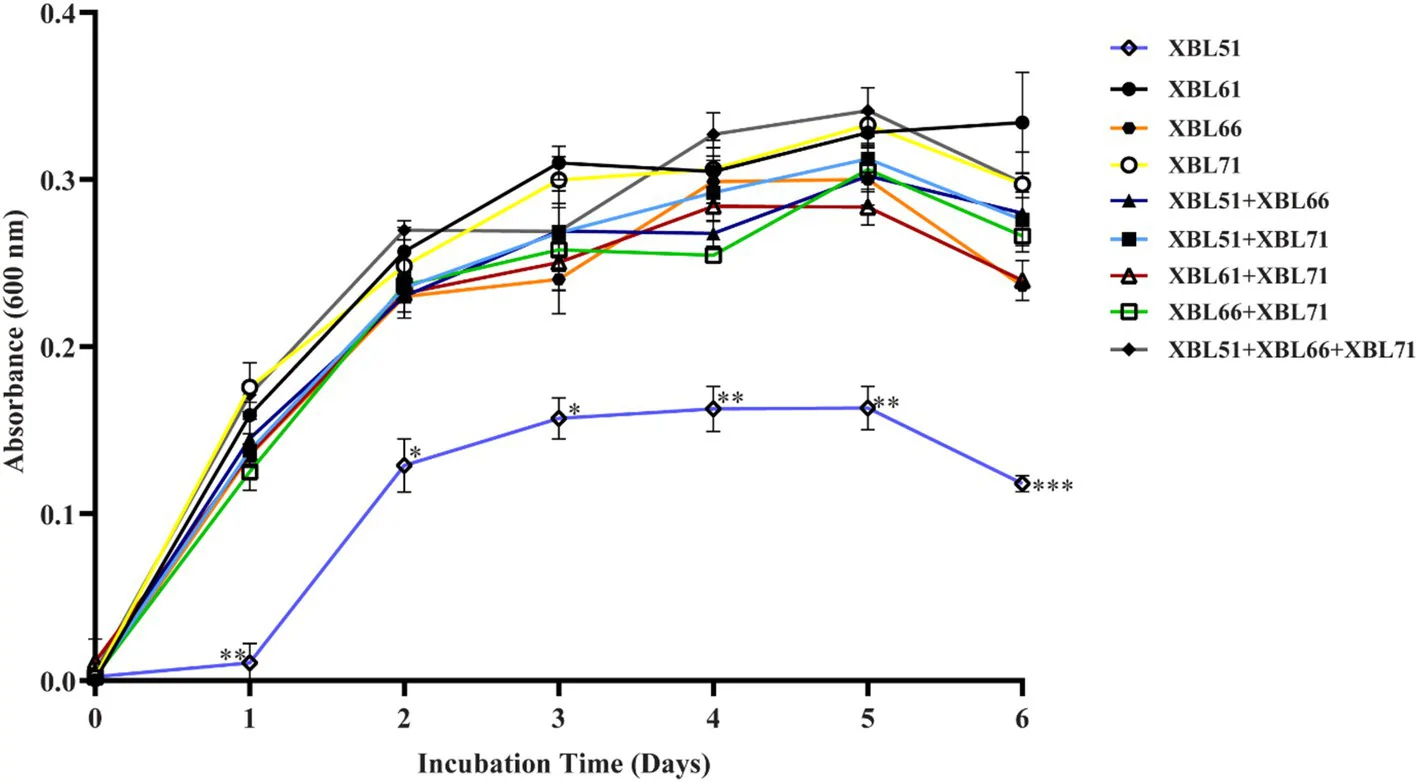
Growth kinetics of individual isolates and microbial consortia on alkaline-pretreated rice husk as sole carbon source under saline-alkaline conditions (4.5% (w/v) NaCl, pH 8.5, 40 °C) for a period of 6 days. Data points represent the mean ± standard deviation of biological replicates. Statistical analysis was performed using a Two-Way Repeated Measures ANOVA followed by Tukey’s multiple comparisons test. Asterisks denote statistically significant reduction in the biomass accumulation of monoculture XBL51 compared to the remaining top-performing isolates and co-cultures across the incubation timeline (*p < 0.05, **p < 0.01, ***p < 0.001).

In most culture conditions, a rapid increase in growth was observed during the first 3 days, which may be mainly due to adaptation and the synthesis of enzymes required to break down readily available complex sugars, such as cellulose and hemicellulose. Further, the growth gradually declines, indicating depletion of reducing sugars in the medium for utilization. As observed in the plate assay, the negative control *E. coli* did not grow in the rice husk-containing minimal media.

In our study, among the various cultures, the XBL61, XBL71 and XBL51 + XBL66 + XBL71 recorded the highest OD_600_ over the 6-day incubation period. Monoculture XBL61 exhibited highly robust growth kinetics characterized by a rapid exponential phase within the first 48 h, followed by continuous biomass accumulation that reached peak optical density on Day 5. Notably, while the majority of other isolates and consortia entered a physiological decline phase by Day 6, XBL61 maintained its peak cellular density. This sustained stationary-phase stability may be due to continuous secretion of extremozymes by the isolate, allowing the efficient degradation and utilization of recalcitrant lignocellulosic biomass over prolonged incubation periods. This hypothesis is supported by the sustained release of reducing sugars observed for XBL61 through Day 4 and Day 5, suggesting ongoing hydrolytic activity even in the later stages of incubation during the 6-day saccharification assay (Supplementary Figure S5).

In case of two-isolate consortium, XBL51 + XBL71 showed the best growth on the rice husk containing media. The initial log phase during Day 1 and 2, followed by the increase in growth upto Day 5, suggested active utilization of the reducing sugars produced by the hydrolysis of the lignocellulosic biomass. This was followed by the decline in growth as observed on Day 6 suggesting the depletion of readily available reducing sugars and the onset of nutrient limitation within the culture medium.

Similarly, the three-isolate consortium (XBL51 + XBL66 + XBL71) exhibited a good growth, suggesting synergistic metabolic interactions among the selected isolates. Recent studies have demonstrated that consortia can efficiently degrade lignocellulosic biomass compared with monocultures due to complementary metabolic activity, in which each microbe produces enzymes that enable cross-feeding among community members (Gad et al., 2024). A similar trend has been observed in a recent study on the degradation of agricultural biomass, where the consortium showed sustained microbial growth compared to individual isolates (Wongfaed et al., 2023). The division of enzymatic labour in a consortium across several species reduces metabolic bottlenecks that often act as a limitation for individual organisms (Liang et al., 2022). Furthermore, in a mixed consortium, the consecutive hydrolysis and consumption of the formed end-products, prevents the saturation of single metabolic pathway leading to efficient utilization of the used biomass (Lin, 2022; Zhu et al., 2016).

On the other hand, XBL51 monoculture showed the least growth. This pattern from near-zero growth on Day 1, followed by evident rise on Day 2 (Figure 3), reflects an extended substrate-adaptation lag characteristic of organisms with tightly regulated inducible hydrolase systems. *Priestia flexa* (XBL51), like many members of the broader *Bacillus* lineage, relies on transcriptional induction of its cellulase and xylanase genes in response to substrate-derived signals, a regulatory mechanism that requires a defined adaptation window for metabolomic reprogramming before enzyme secretion reaches levels sufficient to initiate measurable biomass hydrolysis and support cell division (Bal et al., 2026). The sharp growth transition observed on Day 2 likely indicates the accumulation of extracellular enzymes to release enough reducing sugars from the rice husk to sustain active proliferation of the cells.

The simultaneous reducing sugar and growth kinetic analysis over the 6-day incubation period revealed that monocultures achieved higher saccharification yields as compared to microbial consortia (Figure 4; Supplementary Figure S5). While all the tested halotolerant isolates and consortia showed a high lignocellulosic degradation activity, they employ diverse temporal strategies for biomass hydrolysis. Among all the inoculated isolates, XBL51 + XBL71 showed the highest detectable reducing sugar concentration on Day 1 (1.89 mg/g pretreated rice husk) as indicated in Supplementary Table S5. This initial accumulation of reducing sugars suggests greater synergistic enzymatic hydrolysis of the easily accessible amorphous regions than the early metabolic consumption rate of the culture. However, as the cellular growth of the consortium accelerated on Days 2 and 3, the free reducing sugar concentrations correspondingly declined, reflecting active substrate utilization by the consortium. In contrast, monocultures XBL61 and XBL66 achieved and sustained peak reducing sugar release (up to 1.97 mg/g) through Day 4, indicating the continuous enzymatic depolymerization as well as sustained tolerance to byproducts in the alkaline hydrolysis environment, necessary to support efficient biomass utilization. On the other hand, combinations like the triple consortium (XBL51 + XBL66 + XBL71) yielded lower maximum concentration of reducing sugars, suggesting that resource competition or antagonistic microbial interactions outpaced cooperative hydrolysis (Roell et al., 2019).

**Figure 4:**
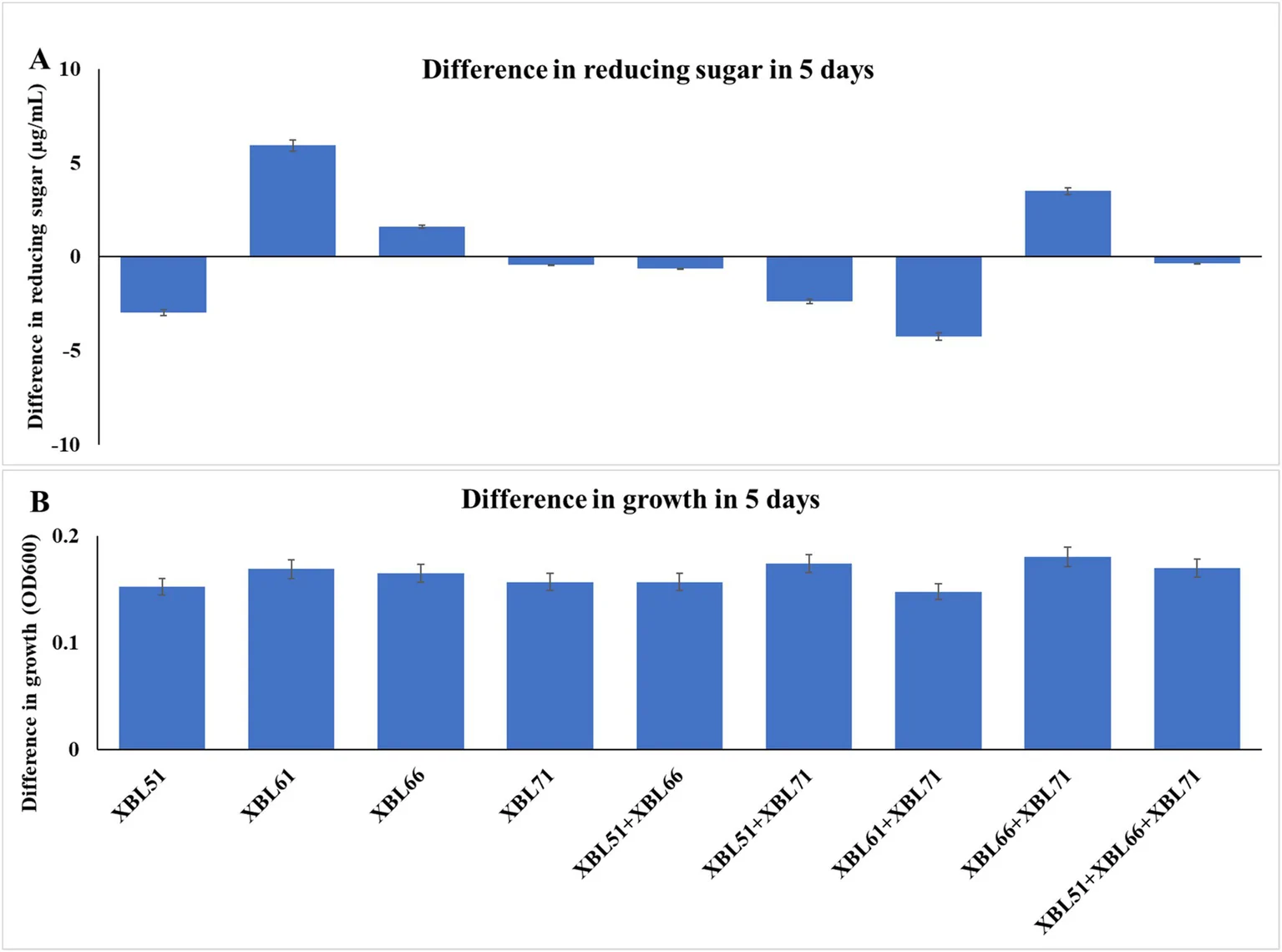
Comparative analysis of reducing sugar utilization and microbial growth among individual isolates and their consortia over a 5-day period. Negative values indicate a net consumption or depletion of reducing sugars from the media, whereas positive values indicate net accumulation or production. **(A)** The bar graph illustrates the net difference in reducing sugar concentration (μg/mL) after 5 days of incubation. **(B)** The corresponding difference in microbial growth over the same 5-day timeframe.

Comparing the net sugar concentrations between day 1 and 5 highlights distinct metabolic patterns exhibited by our isolates. XBL61, XBL66 and XBL66 + XBL71 were the only cultures to show a relative increase in reducing sugars during the 5-day incubation, indicating their sustained hydrolytic stability. Other cultures, including XBL51, XBL51 + XBL71 and XBL61 + XBL71, displayed a sharp decline in the amount of reducing sugars, suggesting rapid cellular assimilation of the available sugars (Figure 4). By day 6, a synchronous decline in detectable sugars was observed across all the samples (Supplementary Figure S5), suggesting the assimilation and bioconversion of the produced sugars into secondary metabolites, consistent with previous reports (Kumar et al., 2025).

Based on the growth kinetics and reducing sugar release analysis, XBL61 monoculture demonstrated exceptional growth kinetics, reaching peak optical density and sustaining dense biomass accumulation through Days 5 and 6 without entering a rapid decline phase. This prolonged viability highlights its ability to sustain higher reducing sugar yields in the later stages of incubation. While the net yield obtained in this study were lower than that of the commercially available solutions, the saccharification achieved under the simultaneous saline-alkaline and thermal stress condition highlight the industrial potential of these polyextremotolerant isolates (Mesbah, 2022).

Furthermore, the XBL51 + XBL71 consortium displayed robust and stable biomass accumulation, coupled with accelerated early-stage saccharification within the first 24 h of incubation compared to other cultures. Although further substrate conversion studies are required to establish the definitive kinetic parameters of this synergistic interaction, these distinct metabolic profiles provided clear targets for further downstream evaluation. Consequently, based on their growth and saccharification efficiencies, we selected XBL61 monoculture (representing sustained sugar utilization) and XBL51 + XBL71 consortium (displaying rapid sugar consumption) for further post-degradation structural analysis using SEM and XRD.

### 3.7 Post-degradation analysis of lignocellulosic biomass

#### 3.7.1 Crystallographic analysis of lignocellulosic degradation

The XRD diffractogram (Figure 5) demonstrates the structural impact of the chemical pretreatment on the raw rice husk. The relative crystallinity of these samples was determined via the crystallinity index (CrI) (Segal et al., 1959), which was calculated as follows:

$$\mathrm{CrI}=\frac{I_{002}-I_{\mathrm{am}}}{I_{002}}\times 100$$

**Figure 5:**
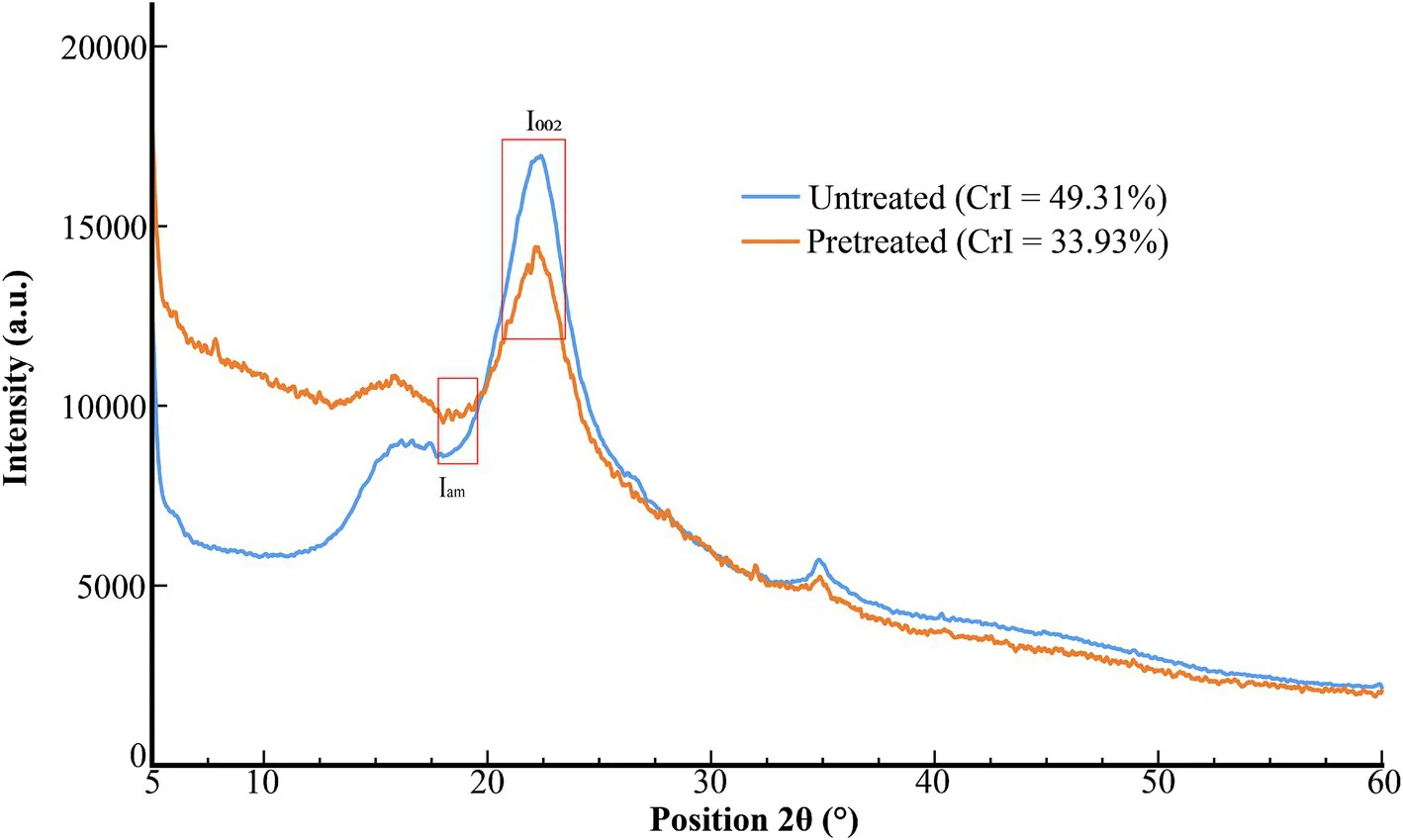
X-ray diffraction (XRD) profiles of untreated and alkali-pretreated (1.5% (*w/v*) NaOH, 60 °C, 90 min) rice husk. I₀₀₂ crystalline peak, 2θ (≥21.5 to ≤23.5°); I_am_ amorphous baseline, 2θ (≥18.0 to ≤19.5°). CrI calculated as [(I₀₀₂ − I_am_)/I₀₀₂] × 100. Elevated background scattering at 2θ < 10° in the pretreated sample is consistent with increased structural disorder and nanoporosity following alkaline pretreatment.

The untreated biomass exhibits a Crystallinity Index (CrI) of 49.31%, reflecting the highly ordered, rigid nature of native cellulose microfibrils bound within the lignin-hemicellulose matrix. Following alkaline pretreatment, the CrI decreases sharply to 33.93%, as indicated by the decline in the crystalline peak (≥21.5 to ≤23.5°). This significant reduction was accompanied by a noticeable elevation in the amorphous region peak. In addition, the diffractogram of the pretreated rice husk reveals a notably elevated scattering intensity at very low 2θ in comparison to the untreated sample (2θ < 10°). This pronounced elevation following alkaline pretreatment likely indicates a substantial increase in the overall amorphous content and extensive structural swelling. Furthermore, given that native rice husk contains approximately 20% silica (Hamidu et al., 2025), this distinct shift may also reflect the partial dissolution and leaching of the protective silica matrix by the NaOH treatment, ultimately suggesting structural loosening and disruption of the recalcitrant biomass (Cheng et al., 2015). While this reduction in the apparent crystallinity index indicates structural deconstruction of the lignocellulosic biomass, it reflects a combinatorial effect involving the partial disruption of crystalline cellulose coupled with dynamic shifts in the relative proportions of cellulose, hemicellulose, lignin and silica following alkaline pretreatment. Together, these compositional and structural alterations create a highly accessible as well as amorphous-rich substrate for subsequent enzymatic hydrolysis and saccharification (Xu et al., 2019).

The XRD diffractogram showing the hydrolyzed samples (XBL61 and XBL51 + XBL71) against the uninoculated control (Figure 6) indicates the dynamic crystallographic shifts that occurred during the 6-day degradation analysis. While the uninoculated control exhibited a crystallinity index of 51.28%, hydrolysis with the XBL61 monoculture resulted in a slight increase in relative crystallinity (CrI = 52.10%). However, the diffractogram demonstrates the reduction in absolute scattering intensity of the crystalline peak (I_002_) for the XBL61-treated sample as compared to the uninoculated control. Thus, suggesting that XBL61 may have contributed to partial degradation of the crystalline cellulose framework, alongside preferential removal of amorphous components. Further, the slight numerical variation of 0.82 in the CrI ratio might have occurred because XBL61 simultaneously hydrolyzed the amorphous regions (I_am_) at a proportionally faster rate than the crystalline regions. This dynamic indicates that during the 6-day incubation, XBL61 might have consumed the highly accessible amorphous fractions (hemicellulose and lignin) while maintaining a simultaneous hydrolytic attack on the crystalline cellulose domains (Oke et al., 2017), resulting in an increased release of reducing sugars, as observed in Supplementary Figure S5.

**Figure 6:**
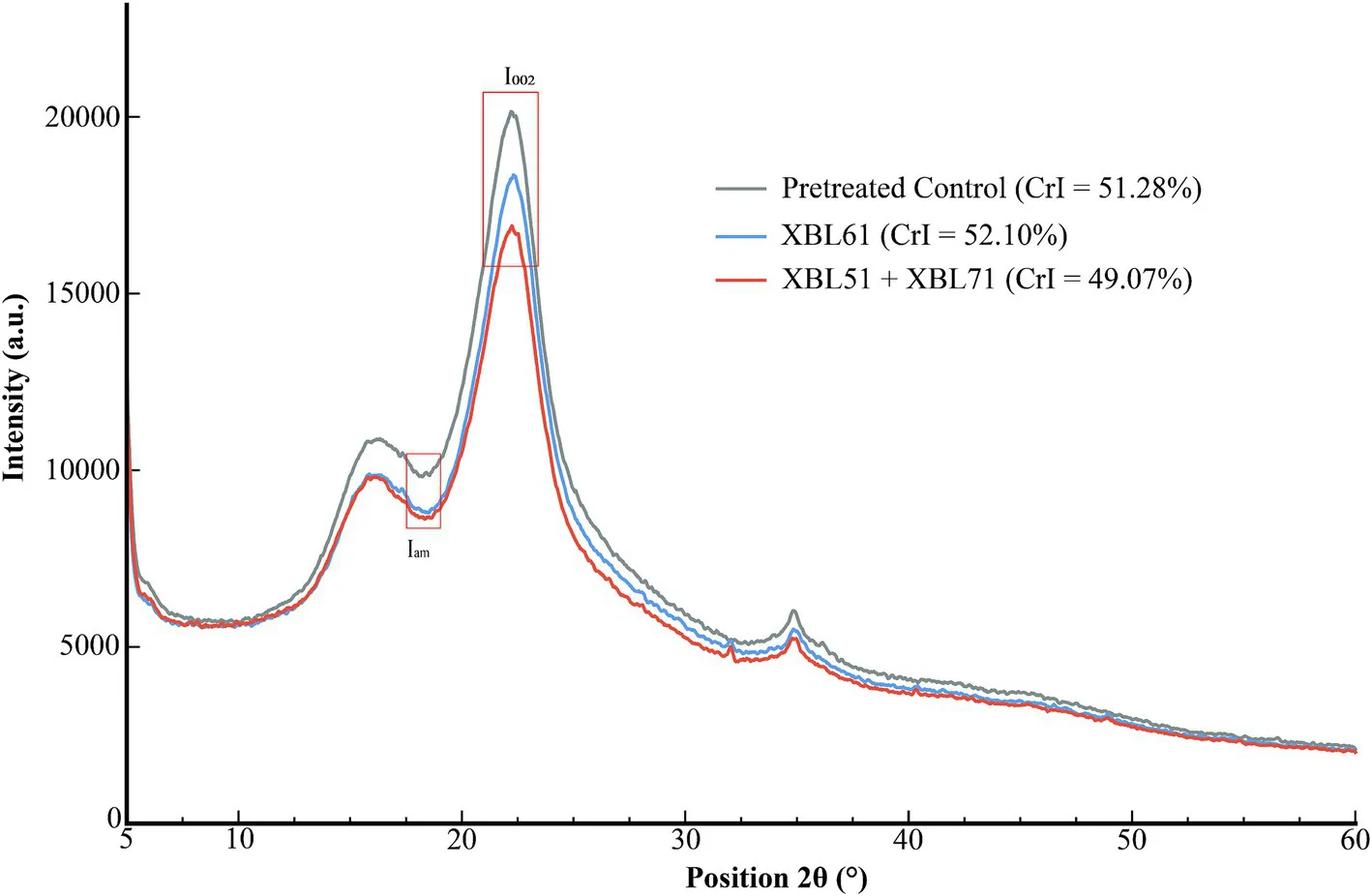
X-ray diffraction (XRD) profiles of uninoculated pretreated control, XBL61-treated, and XBL51 + XBL71 consortium-treated rice husk following 6-day saccharification. Crystallinity index (CrI) values calculated using the Segal method: Control = 51.28%, XBL61 = 52.10%, XBL51 + XBL71 = 49.07%. I₀₀₂ crystalline peak, 2θ (≥21.5 to ≤23.5°); I_am_ amorphous baseline, 2θ (≥18.0 to ≤19.5°). The slight increase in CrI for XBL61-hydrolyzed samples relative to the pretreated control reflects preferential amorphous cellulose degradation, consistent with its high cellulase activity.

In contrast, the XBL51 + XBL71 consortium reduced the CrI to 49.07% compared to 51.28% for the uninoculated control, a shift that indicates enzymatic attack extending beyond the amorphous fraction into the more recalcitrant crystalline regions of the biomass, a broader hydrolytic reach than the individual XBL61 monoculture. These crystallographic differences between XBL61 and the XBL51 + XBL71 consortium are consistent with, and corroborated by, the reducing sugar profiles reported in Section 3.6. Briefly, XBL61 demonstrated increased reducing sugar concentrations, especially at the later stages of the analysis (Day 4 and 5) indicating rapid hydrolysis of the accessible cellulosic and amorphous fractions, releasing reducing sugars in the medium. The XBL51 + XBL71 consortium, on the other hand, maintained an initially accelerated rate of sugar release, followed by hydrolysis and utilization throughout the same period to sustain the growth of both isolates, which in turn required more extensive lignocellulose disruption, as reflected in the greater overall CrI reduction observed in the XRD profiles.

#### 3.7.2 Structural analysis of lignocellulosic degradation

The structural modifications of the rice husk following alkaline pretreatment and subsequent microbial saccharification were further elucidated using Scanning Electron Microscopy (SEM). The untreated raw biomass (Figure 7A) displayed a solid and highly ordered surface characterized by compact, overlapping fibrous lamellae with an intact protective silica-lignin coat. This dense matrix offers minimal accessible surface area, creating a physical barrier that restricts enzymatic access to the internal reducing sugars. Following alkaline pretreatment (Figure 7B), the structural integrity of the biomass was visibly compromised. The surface seems to be flaking, leading to the emergence of longitudinal fissures consistent with the physical loosening of the complex lignocellulosic matrix; which might have caused the rigid outer layers to peel away, exposing the internal holocellulose bundles and drastically increasing the available surface area for subsequent enzymatic attack.

**Figure 7:**
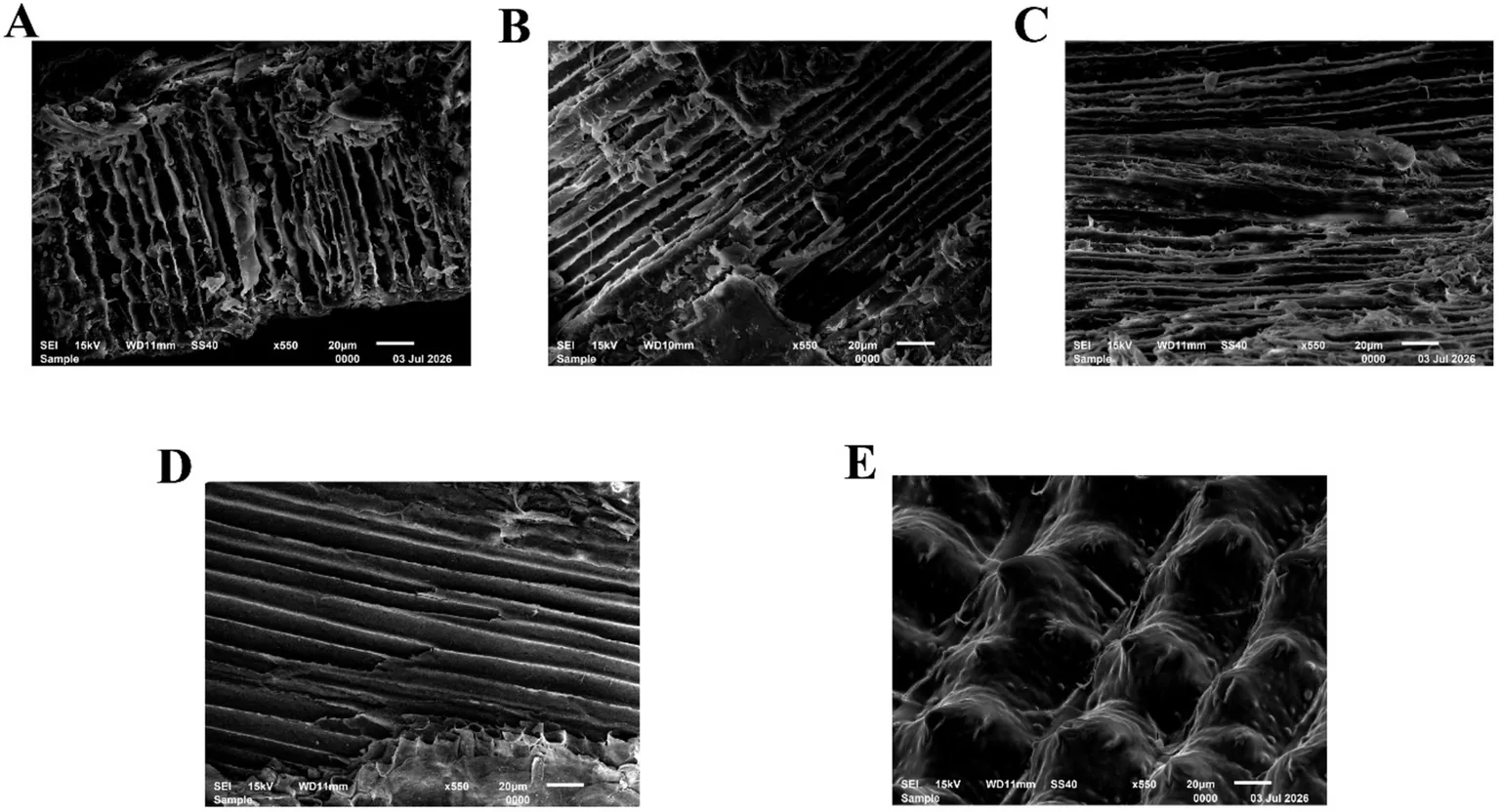
Scanning electron microscopy (SEM) micrographs illustrating the structural morphology of **(A)** Untreated raw biomass; **(B)** Alkaline-pretreated biomass; **(C)** Uninoculated control; **(D)** Biomass hydrolyzed by XBL61; **(E)** Biomass hydrolyzed by XBL51 + XBL71 consortium (550× magnification).

In the case of the uninoculated control (Figure 7C), a defined, parallel fibrous architecture, as observed after pretreatment, was retained during the incubation. On the other hand, lignocellulosic deterioration was clearly exhibited in the hydrolyzed samples. The biomass degraded by the XBL61 monoculture (Figure 7D) displayed significant thinning and grooving of the cellulosic fibers, a physical alteration that visually corroborates its sustained, highly stable saccharification profile. While the hydrolysis carried out through XBL51 + XBL71 consortium (Figure 7E) resulted in extensive structural disruption, with the fibrous lamellae largely disintegrated and replaced by a highly irregular, pitted topography. This structural disruption of the lignocellulosic biomass supports the highly efficient saccharification achieved by our selected isolates, demonstrating the potential of these isolates for application in advanced biofuel-processing (Gu et al., 2018; Singh et al., 2020).

To resolve the extent of structural disruption at the fiber level, higher-magnification (2,700×) SEM imaging was performed on the XBL51 + XBL71 consortium-treated biomass, revealing detail not visible at 550×. The untreated raw biomass (Figure 8A) displayed thick and rigid surface with minimal inherent porosity, demonstrating the highly recalcitrant nature of the native lignocellulosic matrix. Following alkaline pretreatment (Figure 8B), the surface appeared chemically eroded, softer and started to flake into layers. This suggests the successful disruption of the sample’s surface homogeneity, allowing deeper enzymatic access for hydrolysis to take place. However, the most profound structural transformation was observed following saccharification by the XBL51 + XBL71 consortium (Figure 8C). Treatment with the microbial consortia resulted in substantial degradation of the internal cellulosic architecture, transforming the dense fibrous matrix into a highly porous, honeycomb-like framework. This extensive deep-tissue pitting serves as physical evidence of profound structural deconstruction, visually corroborating the enhanced saccharification of the lignocellulosic biomass observed in the biochemical assays. Together, the post-degradation analysis by XRD and SEM indicates that the microbial hydrolysis following alkaline pretreatment of the recalcitrant lignocellulosic waste, results in successful deconstruction of the complex lignocellulose architecture, leading to severe morphological deterioration and decreased crystalline integrity, which ultimately enhances substrate accessibility for microbial hydrolysis to take place (Ramarajan and Manohar, 2017).

**Figure 8:**
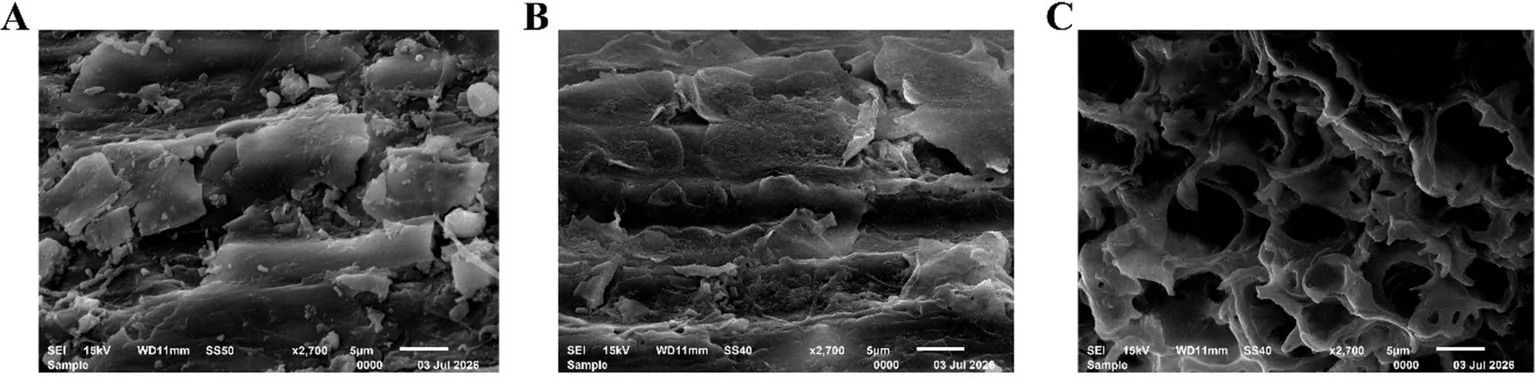
Scanning electron microscopy (SEM) micrographs illustrating the structural morphology of **(A)** untreated raw biomass; **(B)** alkaline-pretreated biomass; **(C)** biomass hydrolyzed by XBL51 + XBL71 consortium (2,700× magnification).

## 4 Conclusion

This study demonstrates the lignocellulolytic potential of polyextremotolerant bacterial isolates and their consortia for the sustainable valorization of lignocellulosic biomass. By coupling targeted alkaline pretreatment with the robust deconstructive capabilities of the halo- and alkali-tolerant isolates, the inherent recalcitrance of rice husk and other related biomass can be successfully overcome. This degradation was demonstrated through structural analysis (SEM) and was accompanied by a significant reduction in the biomass crystallinity index (XRD). The XBL51 + XBL71 consortium demonstrated accelerated early-stage saccharification kinetics, while monocultures of XBL61 and XBL66 showed better sustained yield over the 6-day incubation, highlighting complementary temporal hydrolysis strategies under industrially relevant stress conditions. Ultimately, these polyextremotolerant isolates represent promising candidates for the further development of saline-alkaline lignocellulose bioprocessing. By providing highly efficient saccharification under industrially relevant stress conditions, they establish a critical biological pipeline to advance the global transition toward a circular bioeconomy. The present study establishes a solid biological foundation, from isolation and characterization leading to structural validation of biomass deconstruction, upon which further work can build; the immediate priority should be purification and kinetic characterization of the cellulases and xylanases secreted by XBL61 and the XBL51 + XBL71 consortium under optimized saline-alkaline conditions, followed by coupling the saccharification step with downstream fermentation to quantify actual bioethanol yields from pretreated rice husk, which will determine whether the degradation capacity demonstrated here translates into commercially meaningful conversion efficiencies.

## Data Availability

The datasets presented in this study can be found in online repositories. The names of the repository/repositories and accession number(s) can be found in the article/Supplementary material.

## Footnotes

1
https://www.ezbiocloud.net/

2
http://www.ncbi.nlm.nih.gov/
