# Supplementary material for: Polyextremotolerant lignocellulolytic bacteria from contrasting halo-alkaline habitats as sources of extremozymes for rice husk saccharification and biofuel applications
Source: Front Microbiol. 2026 Oct 28;17:1982443. doi: 10.3389/fmicb.2026.1982443 (PMC13752029; doi:10.3389/fmicb.2026.1982443)
Supplement: Supplementary file 1 [file Supplementary_file_1.DOCX]

**Polyextremotolerant Lignocellulolytic Bacteria from Contrasting Halo-Alkaline Habitats as Sources of Extremozymes for Rice Husk Saccharification and Biofuel Applications**

Anjali Gupta^1^, Shubham Pandey^1^, Anannya Agrawal^1,2^, Ashwini Chauhan^1^, Kuo-Wei Huang^3^*, Ram Karan^1^*

^1^Department of Microbiology, University of Delhi South Campus, New Delhi

^2^Department of Biosciences and Technology, Dr. Vishwanath Karad MIT-World Peace University, Pune

^3^Center for Renewable Energy and Storage Technologies (CREST), KAUST Catalysis Center (KCC), Physical Science and Engineering Division, King Abdullah University of Science and Technology, Thuwal, 23955 Saudi Arabia

*Corresponding author: K.W. Huang (kuowei.huang@kaust.edu.sa); R. Karan (ramkaran@south.du.ac.in)

**SUPPLEMENTARY FILE**

**Table of Contents**

| **Item** | **Title / Description** | **Page No.** |
| --- | --- | --- |
| Supplementary Table S1 | Sampling site coordinates and physicochemical parameters of water and soil samples used for bacterial isolation | 2 |
| Supplementary Table S2 | Morphological characteristics of isolated colonies | 2-3 |
| Supplementary Figure S1 | Colony morphology of representative halotolerant bacterial isolates from three ecologically diverse sampling sites showing distinct morphological characteristics | 3 |
| Supplementary Table S3 | Summary of results of Gram staining and biochemical tests for the bacterial isolates | 4-5 |
| Supplementary Figure S2 | Qualitative enzyme assay plates for xylanase, cellulase, lipase, amylase and protease | 5 |
| Supplementary Table S4 | Zone of hydrolysis produced by enzymatic activity of the bacterial isolates | 5-6 |
| Supplementary Figure S3 | Growth of selected isolates on minimal media plates supplemented with 1% (w/v) rice husk | 6 |
| Supplementary Figure S4 | Compatibility testing between selected isolates for consortia formation | 6 |
| Supplementary Figure S5 | Reducing sugars released by individual isolates and microbial consortia through hydrolysis of alkaline-pretreated rice husk as sole carbon source under saline-alkaline conditions (4.5% (w/v) NaCl, pH 8.5, 40°C) over 6-day incubation | 7 |
| Supplementary Table S5 | Normalized reducing sugar yields (mg/g pretreated rice husk) by polyextremotolerant monocultures and consortia over a 6-day saccharification assay | 7 |

**Supplementary Table S1** Sampling site coordinates and physicochemical parameters of water and soil samples used for bacterial isolation.

| **Location** | **Sample type** | **Colour/ texture** | **pH** | **Chloride concentration**  **(g/L)** |
| --- | --- | --- | --- | --- |
| **Poovar Lake, Kerala** | Water | Clear and Transparent | 6.92 | 1.90 |
| **Varkala Beach, Kerala** | Water | Clear and Transparent | 8.24 | 31.16 |
| **Sanjay Lake, Delhi** | Soil | Brown soil with plant remnants | 9.25 | 0.42 |

**Supplementary Table S2** Morphological characteristics of isolated colonies.

| **Isolate** | **Colour** | **Size** | **Shape** | **Texture** | **Margin** | **Elevation** |
| --- | --- | --- | --- | --- | --- | --- |
| **Poovar Lake Isolates** | | | | | | |
| **XBL52** | Cream | Small | Irregular | Rough | Undulate | Raised |
| **XBL58** | White | Small | Irregular | Smooth | Undulate | Raised |
| **XBL60** | Yellow | Small | Irregular | Smooth | Undulate | Raised |
| **XBL61** | Cream | Small | Irregular | Rough | Undulate | Raised |
| **XBL63** | Creamish white | Small | Circular | Rough | Entire | Raised |
| **XBL64** | White | Large | Irregular | Mucoid | Undulate | Convex |
| **XBL72** | Yellow | Small | Irregular | Smooth | Undulate | Raised |
| **XBL73** | White | Small | Irregular | Mucoid | Undulate | Convex |
| **Varkala Beach Isolates** | | | | | | |
| **XBL53** | Cream with dark centre | Punctiform | Circular | Smooth | Entire | Umbonate |
| **XBL54** | Cream | Medium | Irregular | Mucoid | Undulate | Umbonate |
| **XBL55** | Cream | Small | Irregular | Smooth | Undulate | Convex |
| **XBL71** | Dark cream | Small | Irregular | Mucoid | Undulate | Convex |
| **XBL74** | Creamish white | Medium | Irregular | Mucoid | Undulate | Umbonate |
| **XBL75** | Cream | Medium | Irregular | Mucoid | Undulate | Umbonate |
| **XBL76** | Cream | Small | Irregular | Mucoid | Undulate | Convex |
| **XBL77** | Cream | Small | Irregular | Mucoid | Undulate | Convex |
| **Sanjay Lake Isolates** | | | | | | |
| **XBL51** | Cream | Small | Irregular | Rough | Undulate | Flat |
| **XBL56** | Dark cream | Small | Circular | Smooth | Entire | Raised |
| **XBL57** | Creamish white | Punctiform | Circular | Smooth | Entire | Raised |
| **XBL59** | Orange | Punctiform | Irregular | Rough | Undulate | Flat |
| **XBL62** | Yellowish Cream | Punctiform | Circular | Smooth | Entire | Convex |
| **XBL65** | Creamish white | Small | Circular | Smooth | Entire | Raised |
| **XBL66** | Cream | Small | Irregular | Rough | Undulate | Flat |
| **XBL78** | Yellow | Small | Irregular | Smooth | Undulate | Raised |
| **XBL79** | Creamish white | Small | Circular | Smooth | Entire | Raised |
| **XBL80** | Red | Punctiform | Circular | Mucoid | Entire | Umbonate |
| **XBL81** | Yellowish cream | Punctiform | Circular | Smooth | Entire | Raised |
| **XBL82** | White | Small | Irregular | Smooth | Undulate | Raised |
| **XBL83** | Bright red | Small | Irregular | Smooth | Undulate | Flat |

**Supplementary Figure S1** Colony morphology of representative halotolerant bacterial isolates from three ecologically diverse sampling sites (Poovar Lake, Varkala Beach and Sanjay Lake) showing distinct morphological characteristics.

**Supplementary Table S3** Summary of results of Gram staining and biochemical tests for the bacterial isolates.

| **Isolate** | **Indole test** | **MR test** | **VP test** | **Citrate Utilization test** | **Oxidase test** | **Catalase test** | **Gram staining** |
| --- | --- | --- | --- | --- | --- | --- | --- |
| **Poovar Lake Isolates** | | | | | | | |
| **XBL52** | - | + | - | - | - | + | Gram-positive short rods |
| **XBL58** | - | + | + | + | + | + | Gram-positive rods |
| **XBL60** | - | + | - | - | + | + | Gram-positive long rods |
| **XBL61** | - | + | - | - | + | + | Gram-positive rods |
| **XBL63** | - | + | - | - | - | + | Gram-positive rods |
| **XBL64** | - | - | + | + | + | + | Gram-positive rods |
| **XBL72** | - | + | - | - | + | + | Gram-negative rods in chains |
| **XBL73** | - | - | + | - | - | - | Gram-positive rods in chains |
| **Varkala Beach Isolates** | | | | | | | |
| **XBL53** | - | + | - | - | + | + | Gram-negative rods |
| **XBL54** | - | + | - | - | + | + | Gram-negative cocci |
| **XBL55** | - | - | - | - | + | + | Gram-negative rods |
| **XBL71** | - | - | - | - | - | + | Gram-negative rods |
| **XBL74** | - | + | - | - | + | + | Gram-negative cocci |
| **XBL75** | - | + | - | - | + | + | Gram-negative rods |
| **XBL76** | - | + | - | - | + | + | Gram-negative rods |
| **XBL77** | - | - | - | - | + | + | Gram-negative rods |
| **Sanjay Lake Isolates** | | | | | | | |
| **XBL51** | - | - | - | - | + | + | Gram-positive long rods, have vacuole-like structure |
| **XBL56** | - | + | - | + | - | + | Gram-positive cocci in clusters |
| **XBL57** | + | - | + | + | - | + | Gram-negative short rods |
| **XBL59** | - | - | - | - | - | + | Gram-positive rods |
| **XBL62** | + | + | - | - | - | + | Gram-negative long rods in chains |
| **XBL65** | - | - | + | + | - | + | Gram-negative short rods |
| **XBL66** | - | + | - | + | - | + | Gram-positive long rods |
| **XBL78** | - | - | + | - | - | + | Gram-positive short rods |
| **XBL79** | - | - | + | + | - | + | Gram-positive cocci in clusters |
| **XBL80** | - | - | - | + | - | + | Gram-positive cocobacilli |
| **XBL81** | - | + | - | + | - | + | Gram-negative cocci in clusters |
| **XBL82** | - | + | - | - | - | + | Gram-positive cocci in clusters |
| **XBL83** | - | + | - | - | - | + | Gram-positive coccobacilli |

“+” indicates positive result for the biochemical test, while “-” indicates negative result.

**Supplementary Figure S2** Qualitative enzyme assay plates for (A) xylanase, (B) cellulase, (C) lipase, (D) amylase and (E) protease. The clear halo zone around the colony indicates a positive result for the respective enzyme.

**Supplementary Table S4** Zone of hydrolysis produced by enzymatic activity of the bacterial isolates.

| **Isolate** | **Xylanase (mm)** | **Cellulase (mm)** | **Lipase (mm)** | **Amylase (mm)** | **Protease (mm)** |
| --- | --- | --- | --- | --- | --- |
| **Poovar Lake Isolates** | | | | | |
| **XBL52** | 16.0± 0.3 | - | - | - | 21.1± 0.2 |
| **XBL58** | 32.1± 0.2 | - | - | - | 17.0± 0.1 |
| **XBL60** | - | - | 16.2± 0.4 | - | - |
| **XBL61** | 20.1± 0.1 | 11.5± 0.2 | - | 2.4± 0.1 | 7.1± 0.3 |
| **XBL63** | 21.5± 0.5 | - | - | 7.2± 0.3 | 11.0± 0.2 |
| **XBL64** | 12.0± 0.3 | - | - | 9.2± 0.2 | 14.0± 0.3 |
| **XBL72** | - | - | - | - | - |
| **XBL73** | 24.1± 0.1 | - | - | 8.2± 0.3 | 19.6± 0.4 |
| **Varkala Beach Isolates** | | | | | |
| **XBL53** | 21.0± 0.2 | - | 6.2± 0.2 | 4.1± 0.1 | - |
| **XBL54** | 25.1± 0.3 | - | - | 7.2± 0.4 | 11.2± 0.2 |
| **XBL55** | 17.5± 0.3 | - | 20.2± 0.2 | 4.5± 0.1 | 6.0± 0.1 |
| **XBL71** | 30.2± 0.4 | 17.4± 0.2 | 11.0± 0.3 | 21.1± 0.4 | 7.2± 0.1 |
| **XBL74** | 25.3± 0.3 | - | - | 8.6± 0.1 | 10.2± 0.2 |
| **XBL75** | 28.0± 0.2 | - | - | 4.1± 0.2 | 9.0± 0.1 |
| **XBL76** | 19.5± 0.3 | - | - | 10.2± 0.2 | 7.6± 0.1 |
| **XBL77** | 17.5± 0.2 | - | - | 11.0± 0.1 | 7.2± 0.1 |
| **Sanjay Lake Isolates** | | | | | |
| **XBL51** | 36.0± 0.6 | 6.5± 0.2 | - | 6.0± 0.1 | - |
| **XBL56** | 20.1± 0.4 | - | 15.2± 0.3 | - | 10.4± 0.2 |
| **XBL57** | 10.6± 0.2 | - | - | - | - |
| **XBL59** | - | 12.4± 0.2 | - | 6.8± 0.1 | - |
| **XBL62** | - | - | - | - | - |
| **XBL65** | - | - | - | - | - |
| **XBL66** | 43.5± 0.4 | 26.1± 0.3 | - | 13.4± 0.2 | 19.0± 0.3 |
| **XBL78** | - | - | - | 11.0± 0.2 | - |
| **XBL79** | - | - | - | - | 15.2± 0.2 |
| **XBL80** | - | - | 9.1± 0.1 | - | 11.2± 0.1 |
| **XBL81** | - | - | - | - | 12.0± 0.2 |
| **XBL82** | - | - | - | - | 14.1± 0.5 |
| **XBL83** | - | 14.0± 0.2 | - | - | 28.0± 0.4 |

*Hydrolytic enzyme activities are presented as halo-zone diameters in mm; “-” indicates no detectable activity under the screening conditions.

**Supplementary Figure S3** Growth of selected isolates (A) XBL51, (B) XBL61, (C) XBL66, (D) XBL71 on minimal media plates supplemented with 1% (w/v) rice husk.

**Supplementary Figure S4** Compatibility testing between selected isolates for consortia formation. (A) XBL51 + XBL61 + XBL71- incompatible (inhibition zone circled); (B) XBL51 + XBL66 + XBL71- compatible; (C) XBL61 + XBL66 + XBL71- incompatible (inhibition zone circled).

**Supplementary Figure S5** Reducing sugars release by individual isolates and microbial consortia through hydrolysis of alkaline-pretreated rice husk as the sole carbon source under saline-alkaline conditions (4.5% (w/v) NaCl, pH 8.5, 40°C) over a 6-day incubation. Data represent the mean ± standard deviation (n=3). Statistical significance was determined using a Two-Way Repeated Measures (RM) ANOVA followed by Tukey’s multiple comparisons test. Asterisks denote statistically significant pairwise differences (*p < 0.05). (a) On Day 5, the asterisk indicates a significant difference between monocultures XBL61 and XBL71; (b) On Day 6, asterisks indicate that the XBL61 monoculture demonstrated significantly higher reducing sugar release compared to both the XBL51+XBL71 and XBL61+XBL71 consortia.

**Supplementary Table S5** Normalized reducing sugar yields (mg/g pretreated rice husk) by polyextremotolerant monocultures and consortia over a 6-day saccharification assay.

| **Isolates/ Consortia** | **Reducing Sugar Yields over 6 days (mg/g)** | | | | | |
| --- | --- | --- | --- | --- | --- | --- |
|  | **1** | **2** | **3** | **4** | **5** | **6** |
| XBL51 | 1.88 | 1.79 | 1.74 | 1.82 | 1.80 | 1.83 |
| XBL61 | 1.80 | 1.84 | 1.79 | 1.97 | 1.95 | 1.87 |
| XBL66 | 1.84 | 1.86 | 1.82 | 1.95 | 1.88 | 1.82 |
| XBL71 | 1.73 | 1.79 | 1.78 | 1.78 | 1.72 | 1.69 |
| XBL51+XBL66 | 1.77 | 1.79 | 1.71 | 1.82 | 1.75 | 1.73 |
| XBL51+XBL71 | 1.89 | 1.79 | 1.77 | 1.86 | 1.83 | 1.76 |
| XBL61+XBL71 | 1.85 | 1.79 | 1.73 | 1.77 | 1.74 | 1.74 |
| XBL66+XBL71 | 1.80 | 1.86 | 1.77 | 1.83 | 1.89 | 1.77 |
| XBL51+XBL66+XBL71 | 1.82 | 1.77 | 1.71 | 1.81 | 1.81 | 1.70 |
